# Exploring the Impact of Mitoquinone Supplementation on Glycan Profiles in a Repeated Mild Traumatic Brain Injury Mouse Model

**DOI:** 10.1089/neur.2025.0054

**Published:** 2025-06-16

**Authors:** Mona Goli, Akeem Sanni, Sakshi Gautam, Khalil Mallah, W. Brad Hubbard, Mohammad Reslan, Muhammad Ali Haidar, Karim Halabi, Joseph Walker, Stefania Mondello, Firas Kobeissy, Yehia Mechref

**Affiliations:** ^1^ Department of Chemistry and Biochemistry, Texas Tech University, Lubbock, Texas, USA.; ^2^ Department of Microbiology and Immunology, Medical University of South Carolina, Charleston, South Carolina, USA.; ^3^ Department of Physiology, Spinal Cord and Brain Injury Research Center, University of Kentucky, Lexington, Kentucky, USA.; ^4^ Department of Biochemistry and Molecular Genetics, American University of Beirut, Beirut, Lebanon.; ^5^ Department of Neurobiology, Center for Neurotrauma, Multiomics & Biomarkers, Morehouse School of Medicine, Neuroscience Institute, Atlanta, Georgia, USA.; ^6^ Department of Biomedical and Dental Sciences and Morphological Imaging, University of Messina, Messina, Italy.

**Keywords:** LC-MS/MS, glycomics, neurotrauma, oxidative stress, repeated mild traumatic brain injury

## Abstract

Traumatic brain injury (TBI) represents a significant cause of injury-related deaths and disabilities. Repeated exposure to mechanical impact can lead to metabolic and ionic imbalance, which can cause oxidative stress and worsen the cellular dysfunction initiated by the initial mild TBI (mTBI). Currently, no FDA-approved drug targets repeated mTBI (rmTBI) and its potential sequelae. Mitoquinone (MitoQ) is a mitochondrion-targeted drug that has proven beneficial in different brain-related diseases. We have previously demonstrated the neurotherapeutic effects of MitoQ at a 30-day chronic time point in a similar rmTBI mouse model, where we observed decreased neuroinflammation, enhanced behavioral outcomes, and diminished oxidation. Recently, alterations in glycans have been shown to modulate key roles in the nervous system. Their relevance has been recognized in several neurodegenerative disorders, including TBI, which indicated injury severity and pathobiology. In this study, we aimed to assess brain glycome profiles post MitoQ treatment in experimental rmTBI using liquid chromatography–tandem mass spectrometry. Our findings indicate that there is a correlation between the HexNAc_4_Hex_5_DeoxyHex_3_ glycan profile and MitoQ administration at the acute phase, the levels of HexNAc_4_Hex_4_ glycan in the subacute phase of MitoQ treatment, and the HexNAc_4_Hex_5_ glycan profile at the chronic time point phase of MitoQ treatment. These data suggest that these three glycan profiles can be considered molecular signatures for MitoQ-associated neurotherapy. However, further research is required to validate and establish that these three glycan profiles are accurate and sensitive markers associated with TBI neuroprotection.

## Introduction

Traumatic brain injury (TBI) is one of the most common causes of injury-related disabilities and deaths in young adults.^1^ The Centers for Disease Control and Prevention reported over 69,000 TBI-related deaths in the United States alone in 2021.^2^ TBI has been associated with a number of long-term consequences, including cognitive loss,^3–5^ post-traumatic stress disorder,^6,7^ chronic traumatic encephalopathy,^8,9^ Alzheimer’s disease,^10,11^ and dementia.^12,13^

TBI severity can be categorized as mild, moderate, or severe, with mild being the least severe form. Mild traumatic brain injuries (mTBIs), representing more than 80% of all TBIs, have been associated with neurocognitive and neuropathological impairments.^14^ However, repeated mild TBI (rmTBI) can worsen the symptoms of mTBI and increase the risk of all-cause dementia.^15^ In addition, rmTBI as shown in blast injury and sports concussion has been shown to impact long-term white matter disruption^16^ and increase metabolic and ionic imbalance, leading to oxidative stress.^17–20^ This is because increased oxygen levels and oxygen-derived free radicals, such as hydrogen peroxide, superoxide anions, hydroxyl, and peroxyl radicals, overwhelm the scavenging antioxidant system.^17^ The increased concentration of free radicals can cause alterations in biomolecules such as nucleic acids, proteins, and lipids, thereby affecting cellular processes.^17^ A common theme in altered oxidative stress and cellular bioenergetics relates to mitochondrial dysfunction and impaired bioenergetics, contributing to neural cellular vulnerability, which is observed in several studies of mTBI.^21–24^ Thus, mitochondrial impairment has been proposed as a neurotherapeutic target to ameliorate cellular deficits in metabolic disruption and oxidative stress.^19–22^

Antioxidant drugs have gained attention as neuroprotective agents to ameliorate secondary injury pathways and neurobehavioral deficits.^17,25^ Among these drugs, Mitoquinone Q (MitoQ) is an antioxidant that has been reported to demonstrate neuroprotective antioxidative stress roles in various diseases, including TBI.^17,26–30^ MitoQ can cross the blood–brain barrier (BBB) and cell membranes, accumulate in the mitochondria, and activate the Nrf2/ARE pathway, stimulating antioxidant enzymes.^17^ The translational capabilities of MitoQ are intriguing as it is an over-the-counter supplement and has been shown to exert no long-term adverse effects^17,30^; nevertheless, few studies have investigated the therapeutic efficacy in the area of TBI. In fact, our laboratory has shown that MitoQ exerts neuroprotection in an open head injury at chronic time points,^28^ as well as in rmTBI, in which we showed that MitoQ reduced neuroinflammation and increased the expression of antioxidant proteins.^29^

Along the same lines, proteins have been shown to be major players in mediating pathological mechanisms playing critical roles in biological functions via their post-translational modifications (PTMs).^31^ Several neuroproteomics studies from our laboratory and others have been invested in assessing TBI to identify biomarker profiles^32–37^; however, only a few have focused on PTM alterations as a venue for biomarker strategy. Among these PTMs, protein glycosylation is considered one of the most common PTMs, facilitating various biological processes, including protein stability,^38^ cell signaling,^39^ host–pathogen interactions,^40,41^ and immune functions.^42^ Aberrant protein glycosylation has been associated with many diseases, providing insights into mechanism, progression, and disease state.^43–47^ Furthermore, glycosylation exerts pleiotropic effects on the nervous system, including neuroinflammatory responses, myelination, neuronal excitability, and even impacting neurodegeneration.^48–51^ However, their impact on TBI has not been fully recognized; clinical and pre-clinical studies from our laboratory and others have indicated that glycome-molecular signature can be interrogated as a novel glycan biomarker that can hint and be utilized as indicative of neural injury.^52–54^

In this study, we will assess the glycan profile expression post MitoQ treatment post-rmTBI mouse model at acute (3 days), subacute (7 days), and chronic time points (30 days). We hypothesize that MitoQ administration after rmTBI can modulate glycan profiles related to neural injury/oxidative stress, which may represent critical molecular markers indicative of neurotherapy in the context of rmTBI pathophysiology. This work utilized three mice groups as follows: Sham, rmTBI, and rmTBI + MitoQ cohorts, where rmTBI involved exposure to three closed head injuries administered every 24 h for 3 days. The rmTBI + MitoQ group was administered 5 mg/kg MitoQ twice per week for a month. Cortical brain tissue was used to derive *N*-glycans that were subjected to liquid chromatography–tandem mass spectrometry (LC-MS/MS) analysis to assess the glycan expressions. We conducted qualitative and quantitative analyses of permethylated glycans to improve the structural stability, ionization efficiency, and suitability for reverse-phase liquid chromatography separation.^55–59^

## Methods

### Materials and reagents

Solvents, including high-performance liquid chromatography (HPLC)-grade water, methanol, and acetonitrile (ACN), were acquired from Fisher Scientific (Fair Lawn, New Jersey, USA), and ethanol was purchased from PHARMCO-AAPER. Ammonium bicarbonate (ABC), sodium deoxycholate (SDC), sodium hydroxide (NaOH) beads (20–40 mesh), dimethyl sulfoxide (DMSO, >99.9%), borane–ammonia complex, iodomethane (CH_3_I), and MS-grade formic acid (FA) were purchased from Sigma Aldrich (St. Louis, MO, USA). Peptide:*N*-glycosidase F (PNGase F) was obtained from New England Biolabs (Ipswich, MA, USA).

### Animals

All the procedures performed on animals were approved by the Institutional Animal Care and Use Committees at the American University of Beirut (AUB). Male C57BL/6 mice were obtained from the Animal Care Facility at AUB under the approval number 17–01–458. Mice were group-housed in a temperature-controlled room with a 12-h light/12-h dark cycle and free access to food and water *ad libitum*. At the time of surgery, the mice were 8 weeks old and weighed between 20 g and 25 g. They were divided into three groups as follows: Sham, rmTBI, and rmTBI + MitoQ. For each of the 3 groups, the animals were further divided into 3 subgroups to facilitate animal handling and experimental procedures; one group was sacrificed 3 days after the last surgery (as an acute phase), one group was sacrificed 7 days after the last surgery (as a subacute phase), and another group was sacrificed 30 days after the last surgery (as the chronic time point). All animal experiments were conducted using a randomized block design. Using a computer-generated sequence, mice were randomly assigned to experimental groups (Sham, rmTBI, and rmTBI + MitoQ). In addition, animals were randomly distributed to the experimental groups in a blinded manner, and they were handled by the experimenter for 2 weeks before the commencement of any surgeries. Investigators performing the glycomics analyses and statistical evaluations were blinded to group assignments to reduce potential bias during data acquisition and interpretation.

The number of samples in each phase is as follows: [acute phase: Sham (n = 5), rmTBI (n = 5), rmTBI + MitoQ (n = 4); subacute phase: Sham (n = 5), rmTBI (n = 5), rmTBI + MitoQ (n = 5); chronic time points: Sham (n = 5), rmTBI (n = 5), rmTBI + MitoQ (n = 3)]. For the proteomic profiling component of this study, we utilized five biological replicates per group (n = 5) across three experimental conditions. The experimental design and the procedure timeline are illustrated in Supplementary Figure S1.

### Sample size calculation

This sample size was determined based on a power analysis assuming a one-way ANOVA framework (α = 0.05) with an anticipated large effect size (Cohen’s f ≥0.4), yielding an estimated power of approximately 50–70%. Given the exploratory nature of this study and the typically large proteomic shifts observed in response to neurotraumatic injury, this sample size was considered adequate to capture the most robust and biologically significant protein-level changes. While we acknowledge the limitations in detecting more subtle differences, especially under multiple hypothesis testing corrections, our primary objective was to identify high-confidence candidates for downstream validation. Proteomic data were analyzed with appropriate normalization and false discovery rate control to enhance statistical reliability despite the modest sample size. This design balances ethical considerations, tissue availability, and the need for discovery-driven insights into neurotrauma pathophysiology.

### Repetitive closed head injury

Repetitive closed head injury was performed as described in our previous study.^29^ In brief, this injury model is a modified form of the controlled cortical impact (CCI) aimed to achieve a closed-head impact. While the original protocol of CCI involved a craniotomy operation and the penetration of the brain tissue, a modification was implemented that would allow performing the injury on an intact skull, thus mimicking closed head injuries sustained by sports injury athletes. In the setup, a 5 mm diameter rubber tip was added to the impactor to prevent any damage to the skull. To perform the injury, the mice were anesthetized using a ketamine/xylazine mixture at a dose of 50 mg/kg ketamine and 15 mg/kg xylazine. When the mice reached an unconscious state, as determined by the toe-pinch method, the heads of the mice were fixed in the stereotactic frame using the ear bars. Lubricating eye drops (Xailin®, Nicox, France) were used to prevent their eyes from drying out during the surgery, which could lead to blindness. The head of the mice between the ears was wiped with an iodine solution to prevent any infections; then, a longitudinal incision was made in the middle of the head with a scalpel blade such that the Bregma and Lambda fissures of the skull were apparent. The two fissures were used to guide the impactor tip to the injury site using the Angle Two^TM^ software. The center of the impactor was placed above the somatosensory area of the parietal cortex of the brain (+1.0 mm AP, +1.5 mm ML, and −2 mm DV), which is on the right hemisphere. The impact was made with a velocity of 4 m/s, a depth of cortical deformation of 0.4 mm, and a dwell time of 100 ms. The mouse was then removed from the frame, the incision sutured, and the mouse placed on a heating pad to preserve body temperature. The mouse was allowed to recover while remaining under observation. The injury was performed thrice at each 24-h interval in the 3-day period. The Sham mice were anesthetized and subjected to incisions and suturing for 3 consecutive days without performing the impact.

### MitoQ supplementation

MitoQ (Focus Biomolecules, Plymouth Meeting, PA, USA) was dissolved in 10% DMSO in phosphate-buffered saline (PBS) to a concentration of 25 mg/mL, after which it was diluted with PBS to a working concentration of 0.1 mg/mL. MitoQ was supplemented through intraperitoneal injections at a dose of 5 mg/kg twice per week, starting 1 h after the first injury, then twice weekly for 1 month, as illustrated in Supplementary Figure S1. The 5 mg/kg dosage used is based on published literature and data from our laboratory.^29,41^

### Protein extraction from the brain tissue

Cortical brain tissue samples were initially rinsed with 50 mM ABC buffer. Then, they were mixed with 5% SDC solution with 400 μm molecular biology grade zirconium beads (BMBZ 400–250-36, OPS Diagnostics, LLC) in a 2 mL microtube. The solution of 5% SDC was added to the tissue samples for efficient protein extraction using a bead beater (BeadBug microtube homogenizer, Benchmark Scientific, Edison, NJ) at 4°C. The bead beater was set at 4,000 revolutions/min for 30 sec, followed by a 30-sec pause to cool down. This step was repeated five times. Then, tissue lysate was sonicated in a 0°C ice-water bath for 30 min to improve the protein dissolution. After that, samples were centrifuged at 21,000 *g* for 10 min, and the supernatant was collected and diluted 10 times with 50 mM ABC buffer. The protein concentration was estimated by the BCA Protein Assay Kit (Thermo Fisher Scientific/Pierce, Rockford, IL, USA) following the manufacturer’s protocol.

### Glycan release, reduction, and permethylation

After protein amount estimation, a 200 μg aliquot of extracted proteins from each tissue sample was taken out and diluted with 50 mM ABC buffer to normalize the protein amounts and sample volume. Proteins were then denatured at 90°C for 15 min. After denaturation, glycans were released by PNGase F (3 units of enzyme per 1 μg of protein) and incubated at 37°C for 18 h. After incubation, 2 μL of FA was added to the samples (final concentration 1%) to precipitate the SDC. After thorough centrifugation, the supernatant was dried. Next, 90% ethanol (1 mL) was added to the dried samples and incubated at –20°C for at least 10 min to precipitate proteins. The supernatants, including the glycans, were collected by centrifugation and dried before dialysis. At this stage, the dried samples were dissolved in 50 μL HPLC water and dialyzed against a 500–1000 Da MWCO dialysis membrane to eliminate salts and possible remaining SDC. Samples were then dried, followed by reduction.

The reduction of glycans was followed using the previously reported protocols.^60^ Briefly, 10 μL of fresh reduction solution (10 mg/mL ammonia–borane complex in HPLC water) was added to the dried samples and incubated at 60°C for 1 h. After reduction, an adequate amount of methanol (∼500 μL) was added to the samples and dried to remove the extra borate, which can evaporate together with methanol through the methyl–borate complex. This step was repeated three to five times to eradicate the reduction reagent. The last dried samples were then subjected to permethylation.

**FIG. 1.**
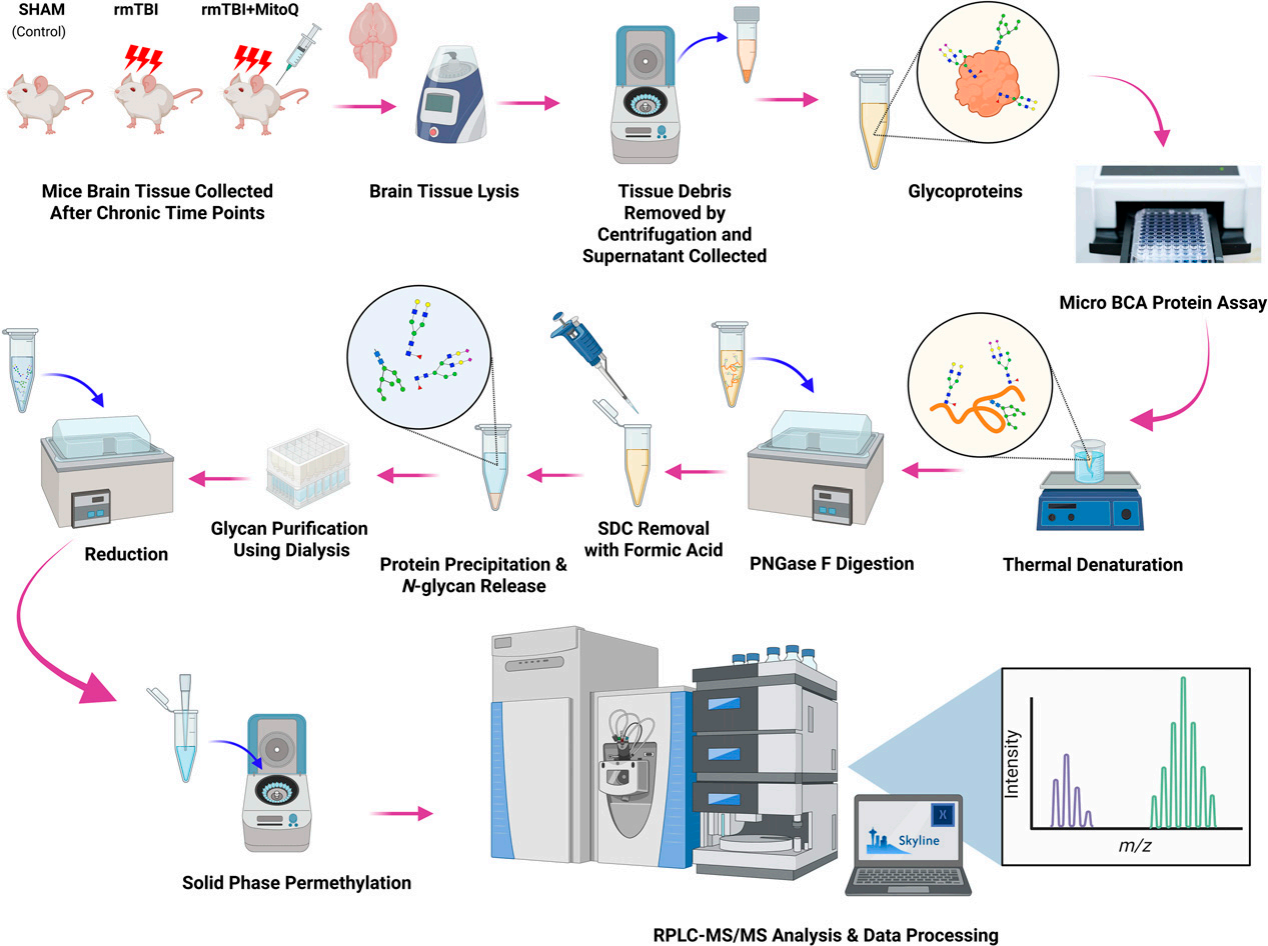
The workflow summarizing sample preparation and glycomics analysis.

The permethylation of the *N*-glycans was performed according to the solid-phase permethylation method.^59^ Initially, sodium hydroxide beads (stored in DMSO) were packed in micro spin columns and rinsed twice with 200 μL DMSO by centrifugation at 1800 rpm for 2 min. Next, dried reduced *N*-glycans were resuspended in 30 μL of DMSO, 20 μL of CH_3_I, and a trace amount of HPLC water (1.2 μL) and loaded into the packed micro spin column. The samples were incubated in the dark for 25 min. After the initial incubation at room temperature, another 20 μL of CH_3_I was added to each sample and incubated for another 15 min. Permethylated glycans were then collected by centrifugation at 1800 rpm for 2 min, followed by further washing with 30 μL of ACN. The collected samples were dried and resuspended in 20% ACN, including 0.1% FA, before LC-MS/MS glycomics analysis. The glycomics workflow is presented in Figure 1.

### Glycomics-based LC-MS/MS analysis

Aliquots of 50 μg PNGase F digest tissue samples derived from the mouse model were subjected to glycomics-based LC-MS/MS analysis. A Dionex 3000 UltiMate NanoLC system (Dionex, Sunnyvale, CA, USA) interfaced with a Q-Exactive HF Orbitrap mass spectrometer (Thermo Scientific, Sunnyvale, CA, USA) equipped with an ESI source was utilized for the analysis. Glycan samples were first loaded to an Acclaim PepMap 100 C18 trap column (75 μm× 2 mm, 3 μm, 100 Å, Thermo Scientific) at a flow rate of 3 μL/min for online desalting. The separation of the *N*-glycans was then accomplished using an Acclaim PepMap 100 C18 capillary column (Acclaim PepMap 100, 75 μm × 150 mm, 2 μm, 100 Å, Thermo Scientific) at 0.35 μL/min at 55°C. Mobile phase A was an aqueous solution of 2% ACN and 0.1% FA, whereas mobile phase B contained 0.1% FA in 98% ACN. The gradient elution of mobile phase B was used as follows: 20% B from 0 to 10 min, 20–42% B from 10 to 11 min, 42–60% B from 11 to 48 min, 60–90% B from 48 to 49 min, 90% B from 49 to 54 min, 90–20% B from 54 to 55 min, and 20% B from 55 to 60 min. The mass spectrometer was set to full-scan positive ionization mode, with a scan range of 700–2000 *m/z*. The full MS resolution was set to 60,000, followed by a dd-MS^2^ scan repeated on the 20 most intense precursor ions. The MS/MS resolution was set at 30,000. The mass accuracy was within 5 ppm, and the normalized collision energy was set to 30%.

### Glycomics-based data processing

Identification of the *N*-glycans in all samples was initially obtained using MultiGlycan software. In MultiGlycan software, experimental *m/z* values of the monoisotopic peaks of ions were searched against the default database to identify the glycan compositions. For the quantitation analysis, Skyline software (MacCoss Lab Software, 64-bit 22.2.0.527) was used to process the obtained raw files from LC-MS/MS based on the transition list generated with MultiGlycan. Skyline quantified each glycan’s peak area by summating all corresponding transition peak areas. Then, all possible *m/z* values of each glycan adduct were evaluated manually.

### Statistical analysis

Statistical analysis and box plots were performed using GraphPad Prism (Version 9.3.1 (350), La Jolla, CA, USA). Principal component analysis (PCA) plots were accomplished in OriginPro 2022 b (64-bit) SR1 9.9.5.171 software (academic version). *N*-glycan compositions were generated using GlycoWorkbench 2, and a workflow scheme was created with biorender.com.

To profile the *N*-glycans in each sample, relative abundance was used to normalize the glycan expressions. Relative abundance was calculated by dividing the peak area of each glycan by the summation area of all identified glycan compositions in each sample. The Kruskal–Wallis test was used to compare the glycan expressions containing more than two groups. Then, Dunn’s test was used to compare the three groups simultaneously. *N*-glycans with *p* values <0.05 were considered significant.

## Results and Discussion

### N-glycome profiles

The *N*-glycome profile of the extracted brain tissue derived from 42 8-week-old mice was acquired by analyzing the reduced and permethylated glycans on a high-throughput C18-LC-MS/MS platform. Glycan profiling data for all cohorts and their average relative abundances at different time points are listed in Supplementary Table S1. In total, 106 *N*-glycans were identified and quantified in different cohorts of our study. In terms of the abundance of different glycan types shown in Supplementary Table S1, we observed that 8 out of the total 106 glycans contained high-mannose structures, accounting for an average of 39% of the total glycan abundance in different cohorts. In contrast, 44 glycans were fucosylated structures, representing an average of 44% of the total abundance in different groups. Five glycans were sialylated, and 39 were sialofucosylated, indicating an average of 1% and 9% of the total abundance in different groups, respectively. Another 10 structures, including nonsialylated and nonfucosylated structures, accounted for an average of 7% of the total abundance in different groups. The relative abundance distribution of different *N*-glycan types is demonstrated in Supplementary Figure S2. Interestingly, the majority of these glycans were high-mannose and fucosylated structures, which is consistent with prior studies.^61,62^ It has been reported that the predominant brain *N*-glycans are high-mannose and fucosylated/bisected structures, and they are less complex in sequence and variety compared with other tissues.^61^ This may be attributed to the lack of extended glycans in the brain, as bisection has been indicated to obstruct subsequent modifications of *N*-glycans, including the addition of galactose and sialic acid.^61,62^ Additional GlcNAc residue may change the glycan conformation to avoid interactions with glycosyltransferases.^61,63,64^

### Unsupervised PCA

**FIG. 2.**
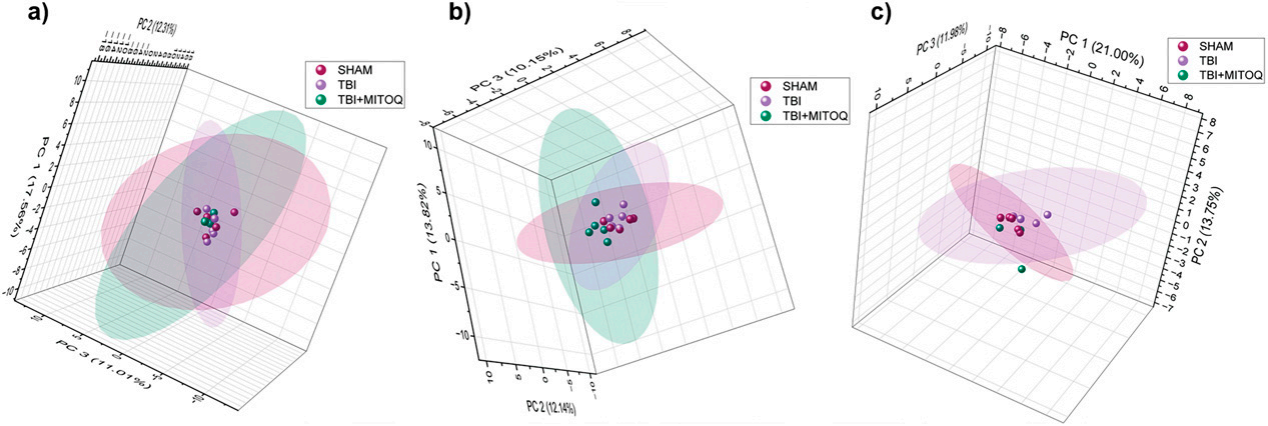
3D Principal component analysis (PCA) of three brain tissue cohorts (Sham, TBI, and TBI + MitoQ) at different stages, **(a)** acute (3 days), **(b)** subacute (7 days), **(c)** chronic time points (30 days) postinjuries based on their glycan relative abundance distributions. Each plot represents one tissue cohort, and the same color denotes the replicates from each cohort. Ellipses represent the 90% confidence levels (CLs). TBI, traumatic brain injury; MitoQ, mitoquinone.

PCA is a mathematical technique that simplifies high-dimensional datasets and turns them into lower dimensional ones using an orthogonal transformation. This generates a set of new variables called principal components (PCs), which can be used to represent patterns of similarity or diversity between observations. Plotting their PCs on a graph shows how far apart two datasets are in terms of PC distance, which indicates their level of variation.^65^ Our study used PCA to determine if different cohorts had different glycan profiles that could distinguish them. We plotted 3D PCA for glycan relative abundances of different brain tissue cohorts at different stages (acute, subacute, and chronic) postinjuries (Fig. 2). The results show that the Sham, TBI, and TBI + MitoQ cohorts are clustered into distinct groups on the PCA plot, indicating different glycan expressions among the groups. For clarification, 2D PCA plots and 3D PCA plots without confidence levels are also provided in Supplementary Figure S3.

### Differentially expressed N-glycans in different cohorts at different time points

Glycosylation is essential for proper neuronal function, influencing the folding, trafficking, and activity of key synaptic proteins and receptors. It is closely tied to glucose metabolism, and disruptions in glycan biosynthesis can impair neuronal signaling and promote endoplasmic reticulum (ER) stress-induced cell death. Glycosylation also modulates neuroinflammation by regulating cytokine signaling and microglial activation through lectin–glycan interactions. Glycan changes amplify inflammatory signaling pathways, contributing to neuronal injury and the progression of neurological disorders.^66^ Alteration in glycan has been reported to play a significant role in the pathophysiology of TBI and spinal cord injury, influencing key processes such as neuroinflammation, BBB disruption, neuronal signaling, and cellular repair mechanisms.^54,67^ Some underlying mechanisms and explanation behind this have been reported.^53,68^

Following TBI, dysregulation of specific glycan structures, including fucosylated glycan, is associated with inflammatory responses.^69^ These modifications enhance the expression of selectin ligands, facilitating leukocyte adhesion and infiltration into the injured brain, thereby contributing to chronic neuroinflammation. Furthermore, glycan changes impact the integrity of the BBB by altering endothelial glycoproteins and the glycocalyx layer, affecting tight junction protein function, and exacerbating vascular permeability.^70^ In neurons and glial cells, TBI-induced glycosylation changes modify the function of membrane-bound receptors, such as neurotrophin receptors (e.g., TrkB), ion channels, and adhesion molecules, thereby influencing synaptic plasticity, axon regeneration, and survival signaling.^71^

Exploring the aberrant glycan profile could offer valuable insights into the molecular aftermath of TBI. Understanding glycan alterations associated with TBI pathology might shed light on potential therapeutic targets. MitoQ, a mitochondrion-targeted antioxidant, has shown promise in mitigating oxidative stress, a hallmark of TBI.^17,28^ Considering the known impact of glycan variations on cellular processes, investigation of the interplay between potential glycan biomarkers and mitochondrial function, possibly influenced by MitoQ, could unveil novel avenues for TBI treatment strategies, bridging the gap between glycan dynamics and mitochondrial resilience in the injured brain.

**FIG. 3.**
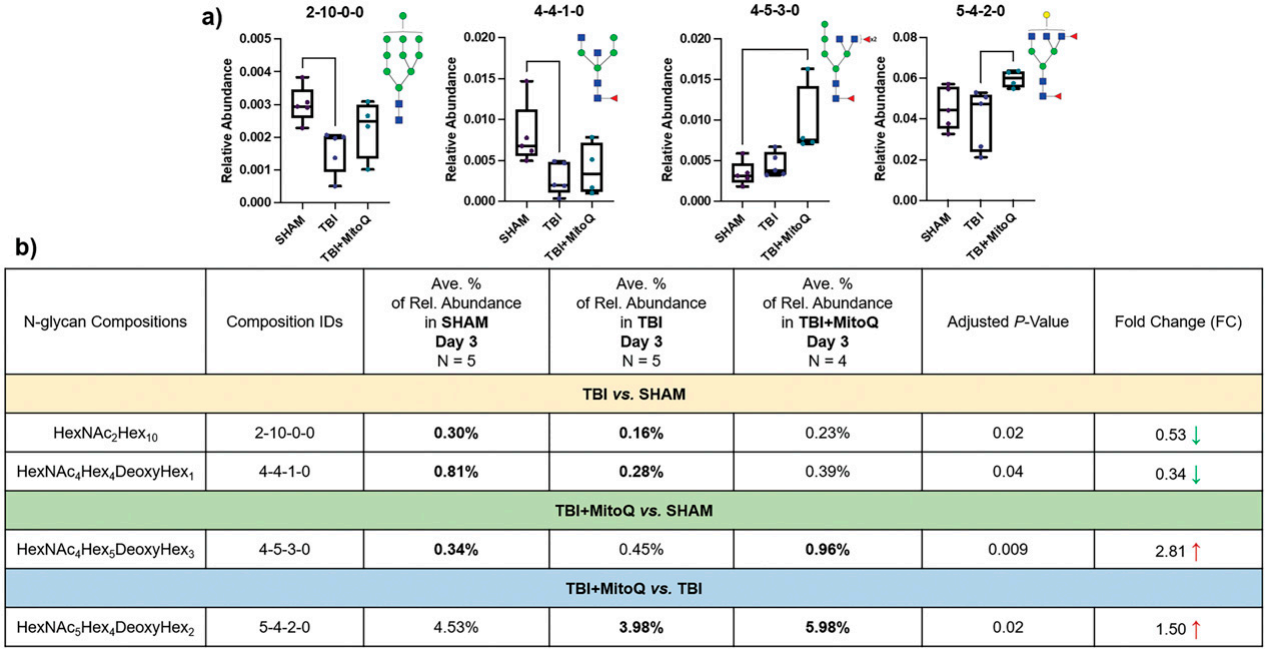
**(a)** Box plots for the relative abundance of *N*-glycans with significant expressions among different tissue cohorts at 3 days’ time postinjury (acute stage). (*denotes *p* value <0.05, **denotes *p* value <0.01). **^†^***N*-glycan code: HexNAc_Hex_DeoxyHex_NeuAc_. **(b)** The list of up- and downregulated *N*-glycans among different tissue cohorts at 3 days’ time points, along with their relative abundances, corresponding *p* values, and fold changes. Symbols: [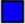], *N-*acetylglucosamine; [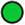], mannose; [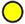], galactose; [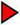], fucose.

In this regard, glycan expression alterations among different cortical brain tissues at different injury phases (acute, subacute, and chronic) were investigated using the Kruskal–Wallis test. Dunn’s multiple corrections were applied to compare the three groups simultaneously (Sham, TBI, and TBI + MitoQ). When looking into the total glycan intensities of three different tissue cohorts at each time point, no significant changes in their total glycan abundances were seen. However, several individual glycan structures showed statistically significant alterations at different time points. In total, four *N*-glycan structures (HexNAc_2_Hex_10_, HexNAc_4_Hex_4_DeoxyHex_1_, HexNAc_4_Hex_5_DeoxyHex_3_, and HexNAc_5_Hex_4_DeoxyHex_2_) exhibited significant expression alterations between different tissue groups (Sham, TBI, and TBI + MitoQ) on acute stage (day 3 postinjuries). The relative abundances of these significant structures were subjected to generate the box plots (Fig. 3a). Our result demonstrated that in the TBI group compared with the Sham group, there was a downregulation of one high-mannose and one fucosylated structure (HexNAc_2_Hex_10_, HexNAc_4_Hex_4_DeoxyHex_1_) with ∼0.53-fold and ∼0.34-fold changes, respectively, among the four abovementioned significant glycans. However, in the TBI + MitoQ group compared with the Sham group, one fucosylated structure (HexNAc_4_Hex_5_DeoxyHex_3_) was upregulated with a ∼2.81-fold change, and the other fucosylated structure (HexNAc_5_Hex_4_DeoxyHex_2_) was upregulated with a ∼1.50-fold change compared with the TBI group. The list of up- and downregulated glycans, along with their relative abundances in different tissue groups at 3-day time points and their corresponding *p* values and fold changes, is presented in Figure 3b. These findings suggest that MitoQ supplementation may potentially affect the regulation of glycans, particularly on fucosylated structures.

**FIG. 4.**
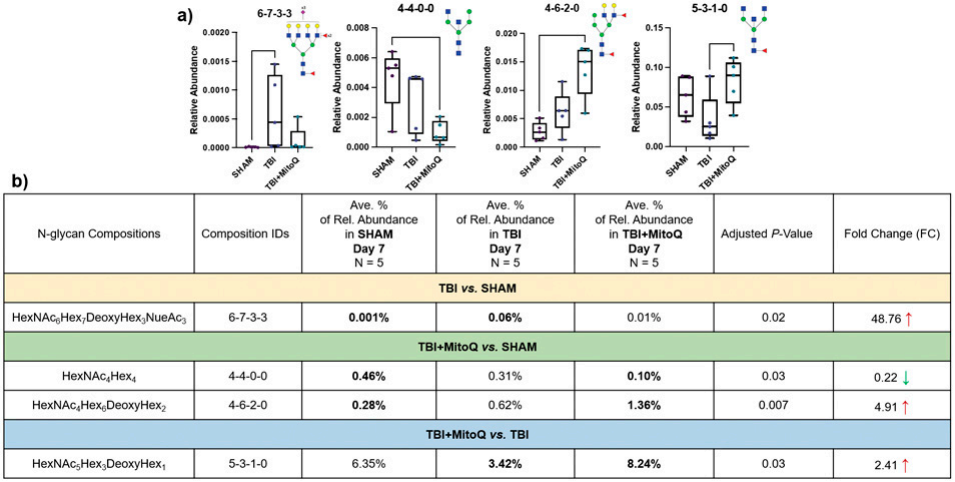
**(a)** Box plots for the relative abundance of *N*-glycans with significant expressions among different tissue cohorts at 7 days’ time postinjury (subacute stage). (*denotes *p* value <0.05, **denotes *p* value <0.01). **^†^***N*-glycan code: HexNAc_Hex_DeoxyHex_NeuAc_. **(b)** The list of up- and downregulated *N*-glycans among different tissue cohorts at 7 days’ time points, along with their relative abundances, corresponding *p* values, and fold changes. Symbols: [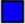], *N-*acetylglucosamine; [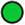], mannose; [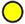], galactose; [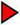], fucose; [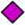], *N-*acetylneuraminic acid.

To study the effect of MitoQ supplementation over the timeline, glycan alteration assessments were also performed on the subacute stage (day 7 postinjuries). Figure 4a illustrates the relative abundances of the four significant glycans in the subacute stage using box plots. As demonstrated here, the relative abundance of one glycan structure (HexNAc_6_Hex_7_DeoxyHex_3_NeuAc_3_) was elevated in TBI groups compared with the Sham group. However, we found two significant glycan alterations when we compared the glycan expression among TBI + MitoQ and Sham groups. Among them, HexNAc_4_Hex_4_ was downregulated and HexNAc_4_Hex_6_DeoxyHex_2_ was upregulated in TBI + MitoQ with fold changes of ∼0.22 and ∼4.91, respectively. In contrast, no significant alterations in glycan expression were observed between the TBI + MitoQ and TBI cohorts, except for the HexNAc_5_Hex_3_DeoxyHex_1_ structure, which is fucosylated and was enhanced in TBI + MitoQ with a fold change of ∼2.41. Figure 4b denotes the relative abundances of these significant glycans along with their corresponding *p* values and fold changes.

**FIG. 5.**
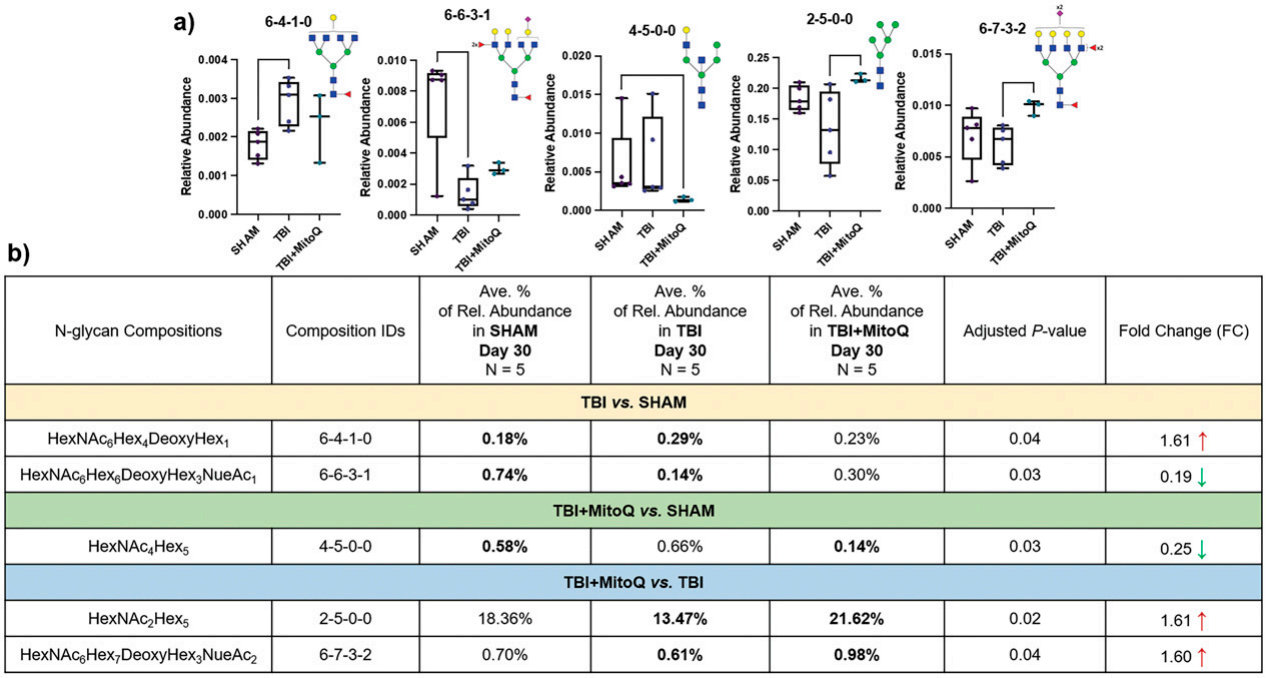
**(a)** Box plots for the relative abundance of *N-*glycans with significant expressions among different tissue cohorts at 30 days’ time postinjury (chronic time points). (*denotes *p* value <0.05, **denotes *p* value <0.01). **^†^***N*-glycan code: HexNAc_Hex_DeoxyHex_NeuAc_. **(b)** The list of up- and downregulated *N*-glycans among different tissue cohorts at 30 days’ time points, along with their relative abundances, corresponding *p* values, and fold changes. Symbols: [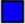], *N-*acetylglucosamine; [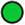], mannose; [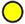], galactose; [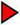], fucose; [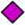], *N-*acetylneuraminic acid.

When we assessed the glycan expressions among different tissue groups (Sham, TBI, and TBI + MitoQ) at the chronic time point (30 days postinjuries), we found that one fucosylated structure (HexNAc_6_Hex_4_DeoxyHex_1_) was upregulated and one sialofucosylated structure (HexNAc_6_Hex_6_DeoxyHex_3_NeuAc_1_) was downregulated in the TBI groups compared with Sham, with the fold changes of ∼1.61 and ∼0.19, respectively. Conversely, we found that the relative abundance of the HexNAc_4_Hex_5_ glycan structure was lower-expressed ∼0.25 times in the TBI + MitoQ group compared with Sham, whereas the relative abundances of one high-mannose structure (HexNAc_2_Hex_5_) and one sialofucosylated structure (HexNAc_6_Hex_7_DeoxyHex_3_NeuAc_2_) were overexpressed ∼1.61 and ∼1.60 times, respectively, versus the TBI cohort. Figure 5a demonstrates the relative abundances of the five significant glycans on chronic time points using box plots. In addition, the list of up- and downregulated glycans in our pairwise comparisons among different groups on chronic time points (day 30 postinjuries), along with their relative abundances and their corresponding *p* values and fold changes, is also indicated in Figure 5b.

To further examine the effect of MitoQ on glycan expressions and their correlations across different groups and stages in the repeated mild TBI mouse model, we have included bar graphs that compare 16 individual glycan structures with significant expressions in Supplementary Figures S4–S7, categorized based on the glycan types. Our findings provide evidence for the potential of MitoQ to alleviate glycan alterations following injury. As can be observed in Supplementary Figures S4–S7, in total, 16 out of 106 identified glycan structures showed significant expression in any possible pairwise comparisons. Among these 16 *N*-glycan structures with significant expressions, the HexNAc_4_Hex_4_, HexNAc_4_Hex_4_DeoxyHex_1_, HexNAc_4_Hex_5_, HexNAc_4_Hex_5_DeoxyHex_3_, HexNAc_5_Hex_3_DeoxyHex_1_, HexNAc_5_Hex_4_DeoxyHex_2_, and HexNAc_6_Hex_4_DeoxyHex_1_ structures have been confirmed as brain tissue-specific structures, consistent with other literature^61,62^ and the Consortium for Functional Glycomics’s website (http://www.functionalglycomics.org).

Our findings show that the majority of the glycans with significant expression at different phases are high-mannose and fucosylated glycan structures. There could be various factors responsible for these observations. Given that glycosylation is highly influenced by the physiological state of cells and is specific to cell type and site,^68^ it is possible that the alterations noticed in our mouse model brain tissue glycans result from the metabolic and biosynthetic challenges that occur post-TBI. Following an acute brain injury, the neurons undergo mitochondrial dysfunction along with elevated oxidative stress, leading to a shift from aerobic to anaerobic metabolism, and this could be one of the mechanisms contributing to the changes in specific glycan types.^53^ In addition, according to the literature, it has been noted that the turnover rates of high-mannose *N*-glycans increase under oxidative stress conditions,^72^ and it is suggested that core fucose expression may also be enhanced during the antioxidant response.^73^

After acute brain injury, a series of interconnected mechanisms disrupt mitochondrial function and increase oxidative stress, forcing neurons to transition from aerobic to anaerobic metabolism.^74,75^ As glucose and glutamine are diverted into glycolysis and glutaminolysis, the hexosamine biosynthetic pathway is deprived of its substrates, leading to decreased production of UDP-GlcNAc—a key molecule required for *N*-glycan biosynthesis in the Golgi apparatus. This disruption results in abnormal glycosylation patterns.^76–78^ These glycan changes serve as biochemical signatures of oxidative injury and can be detected in both brain tissue and circulating biofluids. Notably, disruption in the expression of specific glycan structures has been strongly linked to impaired cell development, enhanced pro-inflammatory cell differentiation, and compromised mitochondrial health.^77^

In addition, aberrant glycosylation contributes to neuroinflammation by altering the structure and function of cytokines, chemokines, and their receptors, thereby enhancing immune cell activation through lectin-mediated pathways involving siglecs, galectins, and mannose-binding lectins (MBLs). This results in elevated production of pro-inflammatory mediators such as IL-1β, TNF-α, and reactive oxygen species (ROS), perpetuating chronic inflammation in the injured brain.^66^ Altered glycosylation enhances neuroinflammation by modifying the structure and function of cytokines, chemokines, and their receptors. Glycan changes influence immune cell activation via lectin interactions (e.g., with siglecs, galectins, and MBLs), leading to heightened release of pro-inflammatory mediators such as IL-1β, TNF-α, and ROS.^66^ In this way, aberrant glycosylation amplifies innate immune responses and sustains chronic inflammation in the injured brain.

Disruptions in glycosylation compromise the unfolded protein response, disrupt ER homeostasis, and impair the recycling of cell-surface receptors, collectively hindering neurogenesis, axonal regeneration, and synaptic remodeling.^79–81^ Consequently, restoring glycosylation homeostasis may enhance cellular resilience and promote functional recovery following neural injury. Importantly, supplementation with MitoQ, a mitochondrion-targeted antioxidant, demonstrates potential efficacy in attenuating oxidative stress and restoring normal glycosylation patterns. Our findings highlight the intricate relationship between the high-mannose/fucosylated structures and oxidative stress markers, suggesting a possible mechanism underlying cellular dysfunction and TBI progression.

In addition, it has been reported that the high levels of HexNAc_4_Hex_5_, HexNAc_4_Hex_4_, and HexNAc_6_Hex_3_DeoxyHex_1_ and low levels of HexNAc_5_Hex_5_DeoxyHex_3_, HexNAc_5_Hex_6_DeoxyHex_3_NeuAc_1_, and HexNAc_4_Hex_5_DeoxyHex_3_ observed in key brain regions of the prefrontal cortex, hippocampus, olfactory bulb, and cerebellum are associated with the segregation trend.^62^ This distinctive pattern suggests potential implications for glycan-mediated processes in these regions, prompting further exploration into the functional significance of these specific glycans in neural mechanisms. This study suggests a correlation between the HexNAc_4_Hex_5_DeoxyHex_3_ glycan profile and MitoQ treatment in the acute stage, HexNAc_4_Hex_4_ glycan profile and MitoQ treatment in the subacute stage, and HexNAc_4_Hex_5_ glycan profile and MitoQ treatment in the chronic time points. Remarkably, the introduction of MitoQ treatment following TBI appears to modulate the segregation pattern associated with these specific glycans, indicating a potential influence on cellular responses. Understanding the spatial variations of these glycans may provide insights into their roles in neuronal functions and contribute to our comprehension of glycan-related dynamics within the nervous system and the effect of MitoQ on TBI neurotherapy.

Our research findings shed light on the intricate relationship between oxidative stress, glycan dysregulation, and rmTBI, emphasizing the crucial role of glycan-mediated neuroinflammation in TBI pathogenesis. Furthermore, the efficacy of MitoQ supplementation in mitigating TBI-induced glycan expression changes suggests a promising therapeutic approach for alleviating neuroinflammatory responses and enhancing recovery postinjury. These insights deepen our understanding of TBI mechanisms and offer potential avenues for targeted interventions to improve outcomes in TBI patients.

## Conclusion

rmTBI observed in contact sports athletes, as well as in military personnel, represents a major health concern leading to neuropsychological deficits and early occurrence of neurodegenerative disorders without an FDA-approved therapy.^82^ Among the neuropathological features of the secondary injury phase are the altered bioenergetics and oxidative stress attributed to mitochondrial dysfunction post-mTBI, making this an attractive target for therapy.^19–22^ Since MitoQ, an over-the-counter supplement, has shown its neuroprotection via combating oxidative stress targeting the mitochondria, it represents an optimal candidate to test in the context of TBI.^83,84^ In this work, which is a continuation for our previous study, we demonstrated that the same paradigm of administering MitoQ post-rmTBI exhibited neurobehavioral and neurocognitive rehabilitation, decreased neuroinflammation, as well as elevated antioxidative enzymes, at the chronic time point of 30 days.^29^ Here, a neuro-glycomics approach was conducted to investigate the glycan alteration profile in the MitoQ-treated mice with TBI.

Our LC-MS/MS analysis investigation into glycan profiling in a TBI mouse model has illuminated the intricate molecular landscape underlying TBI-induced changes. The comprehensive glycomics analysis provided valuable insights into the dynamic alterations of glycans, shedding light on potential biomarkers involved in TBI pathology. Furthermore, our study explored the therapeutic potential of MitoQ in ameliorating glycan dysregulation post-TBI. The observed effects of MitoQ on mitigating glycan alterations (such as HexNAc_2_Hex_10_, HexNAc_4_Hex_4_DeoxyHex_1_, and HexNAc_5_Hex_4_DeoxyHex_2_ in the acute stage, HexNAc_5_Hex_3_DeoxyHex_1_ and HexNAc_6_Hex_7_DeoxyHex_3_NeuAc_3_ in the subacute stage, and HexNAc_2_Hex_5_, HexNAc_6_Hex_4_DeoxyHex_1_, HexNAc_6_Hex_6_DeoxyHex_3_NeuAc_1_, and HexNAc_6_Hex_7_DeoxyHex_3_NeuAc_2_ in chronic time points) suggest a promising avenue for intervention, with implications for developing targeted strategies to modulate the glycomics response and improve outcomes in TBI patients.

It is worth mentioning that although putative glycan markers were identified and quantified in response to MitoQ supplementation in this study, additional supportive validation could be obtained through the targeted glycan analyses and ELISA assays’ design to quantify the *N*-glycans of interest. Besides, the investigation of the MitoQ supplement on the glycan isomer expression in our rmTBI mouse model would provide more comprehensive data. These findings underscore the importance of glycan profiling as a powerful tool in unraveling the complexities of TBI pathology and highlight the potential of MitoQ as a therapeutic agent. The integration of glycan analysis with targeted interventions not only enhances our understanding of TBI at the molecular level but also opens new avenues for precision medicine approaches in treating TBI. As we navigate the intricacies of glycan dynamics and therapeutic modulation, our study contributes to the growing body of knowledge aimed at advancing TBI research and fostering the development of innovative strategies to enhance recovery and minimize the long-term impact of TBI.

## Data Availability

The raw data generated from LC-MS/MS have been submitted to GlycoPOST with accession number GPST000399.

## Abbreviations Used

ABC: ammonium bicarbonate
ACN: acetonitrile
AUB: American University of Beirut
BBB: blood–brain barrier
BMBZ: biology grade zirconium beads
CCI: controlled cortical impact
CH_3_I: iodomethane
CL: confidence level
DMSO: dimethyl sulfoxide
ER: endoplasmic reticulum
FA: formic acid
HPLC: high-performance liquid chromatography
LC-MS/MS: liquid chromatography–tandem mass spectrometry
MBLs: mannose-binding lectins
MitoQ: Mitoquinone
mTBI: mild traumatic brain injury
NaOH: sodium hydroxide
PBS: phosphate-buffered saline
PCA: principal component analysis
PCs: principal components
PNGase F: Peptide:*N*-glycosidase F
PTMs: post-translational modifications
rmTBI: repeated mild traumatic brain injury
ROS: reactive oxygen species
SDC: sodium deoxycholate
TBI: traumatic brain injury
