# Supplementary material for: Exploring the Impact of Mitoquinone Supplementation on Glycan Profiles in a Repeated Mild Traumatic Brain Injury Mouse Model
Source: Neurotrauma Rep. 2025 Jun 16;6(1):525–38. doi: 10.1089/neur.2025.0054 (PMC12237849; doi:10.1089/neur.2025.0054)

SUPPLEMENTARY INFORMATION

**Supporting Information Figure S1**

**Figure S1. Timeline of the Experimental Design**. The mice were divided into Sham, TBI, and TBI+MitoQ cohorts. The TBI and TBI+MitoQ cohorts were subjected to mild TBI on three consecutive days. Following the first mild TBI injury, MitoQ (5 mg/kg) was administered to the TBI+MitoQ group and then twice per week over a month. On days 3 (acute phase), 7 (subacute phase), and 30 (chronic time points) after the last mild injury, the mice were sacrificed, and the tissues were extracted for molecular assessments. Number of samples in each phase is as follows: [acute phase: sham (n = 5); rmTBI (n = 5), rmTBI + MitoQ (n = 4); subacute phase: sham (n = 5); rmTBI (n = 5), rmTBI + MitoQ (n = 5); chronic time points: sham (n = 5), rmTBI (n = 5), rmTBI + MitoQ (n = 3)].


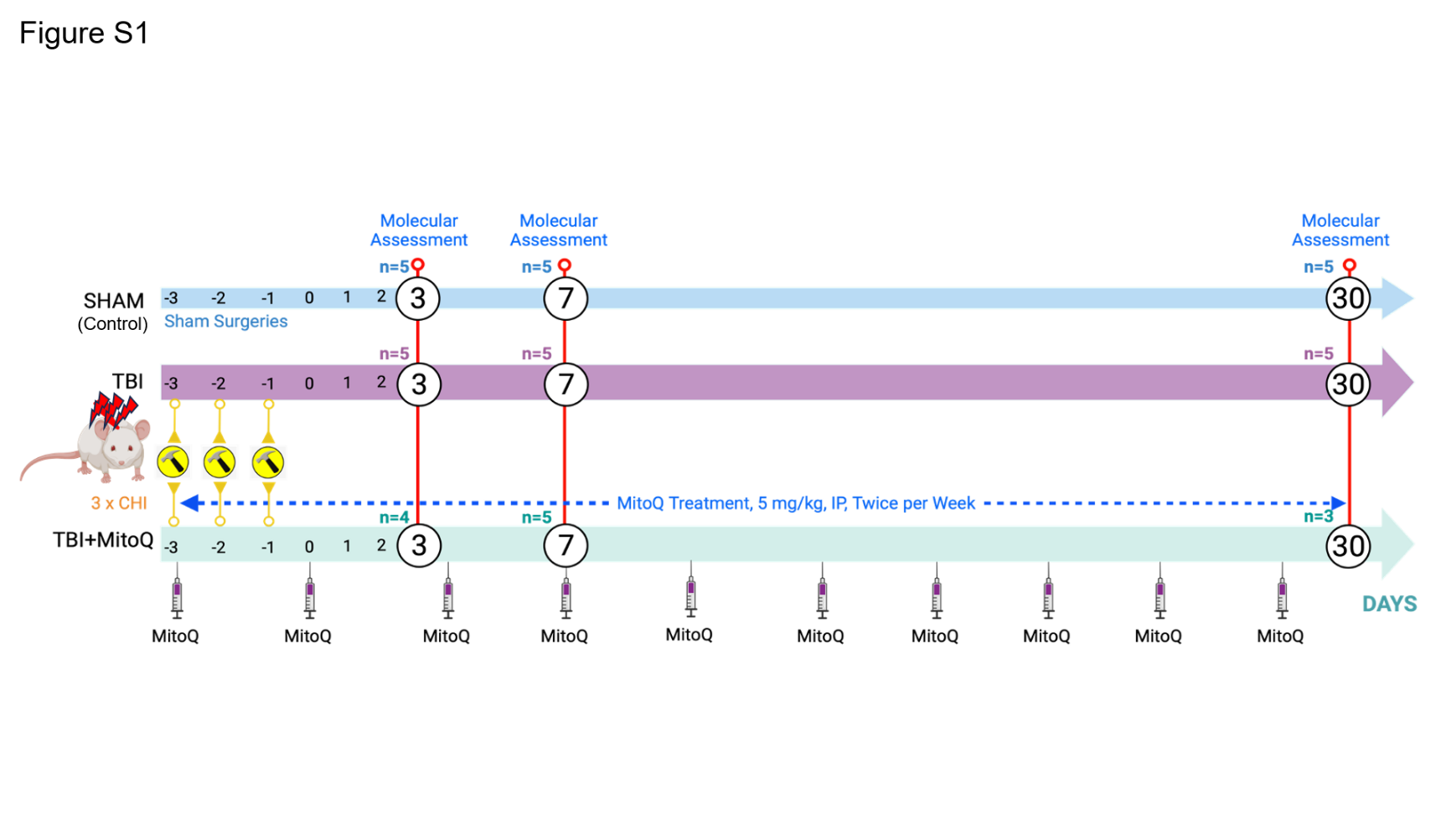


**Supporting Information Figure S2**

**Figure S2.** **The relative abundance distribution of different *N*-glycan types in different tissue cohorts** at (**a**) 3 Days (acute stage), (**b**) 7 Days (subacute stage), and (**c**) 30 Days (chronic time points). The majority of these glycans are high-mannose and fucosylated structures.


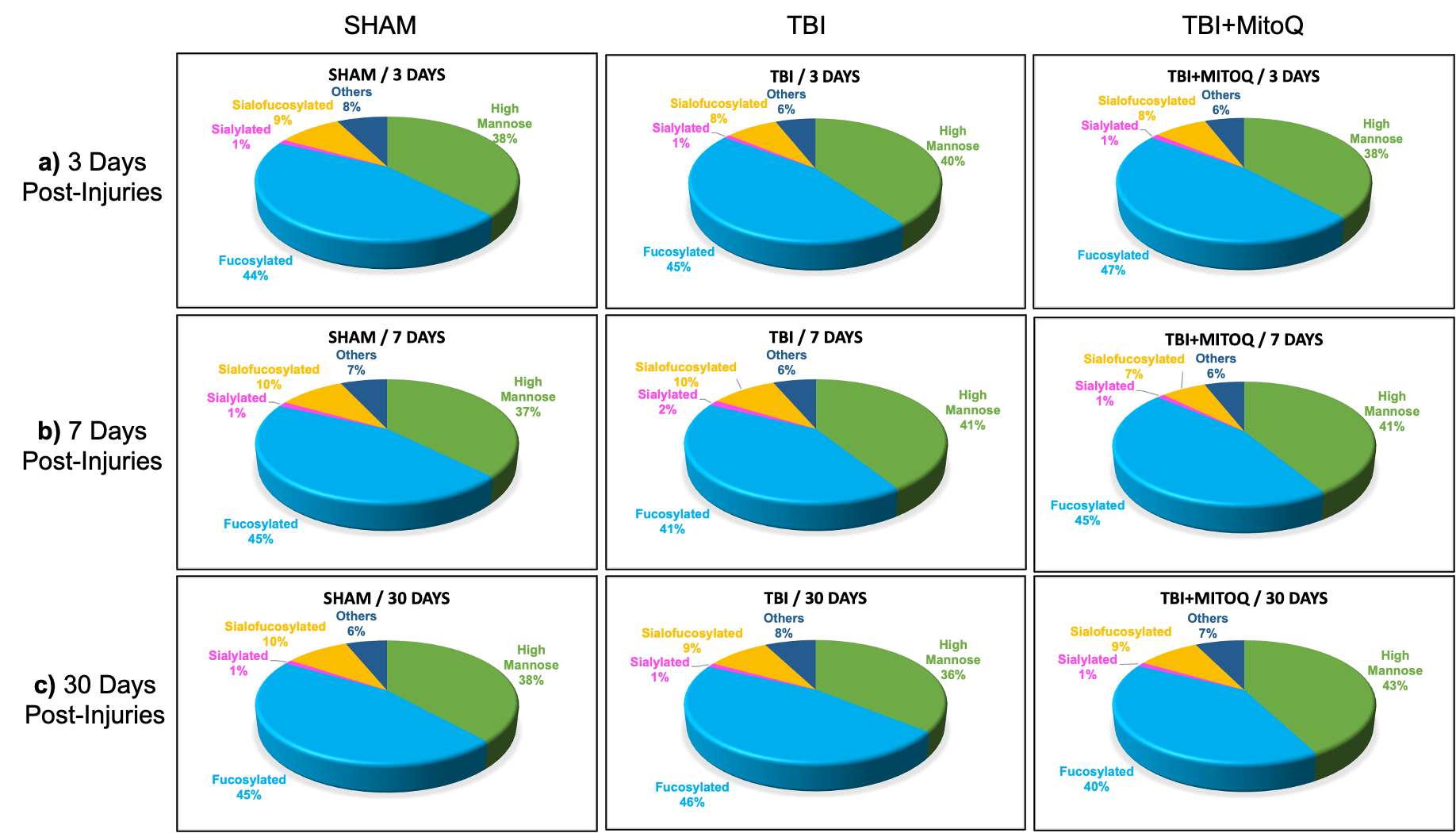


**Supporting Information Figure S3**

**Figure S3.** (**i**) **2D Principal component analysis (PCA) of three brain tissue cohorts (SHAM, TBI, and TBI+MitoQ) at different stages**, (**a**) acute (3 Days), (**b**) subacute (7 Days), (**c**) chronic time points (30 Days) post-injuries based on their glycan relative abundance distributions. Each plot represents one tissue cohort, and the same color denotes the replicates from each cohort. Ellipses represent the 90% confidence levels (CLs). (**ii**) 3D PCA of the same cohorts without considering the CLs.


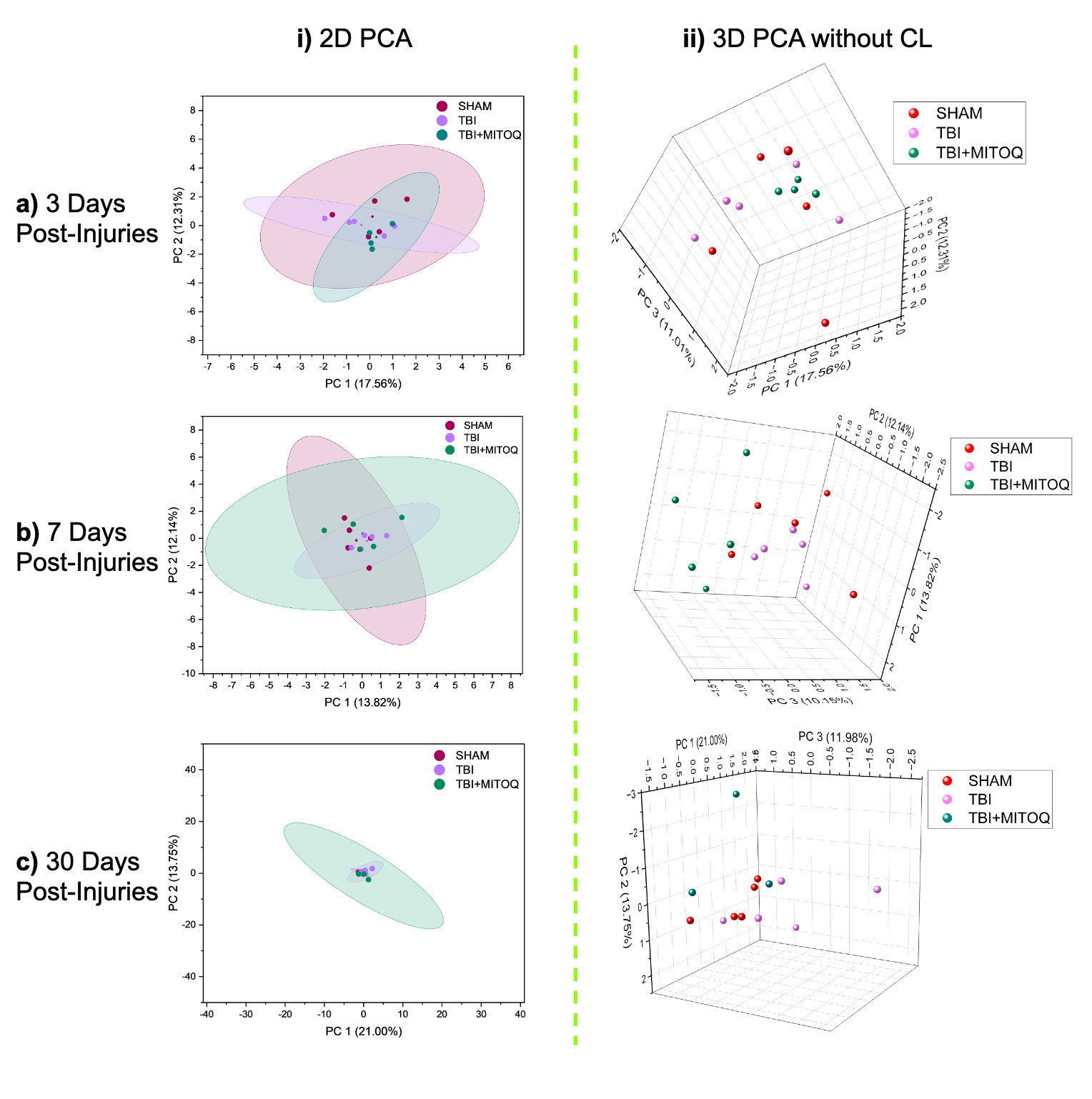


**Supporting Information Figure S4**

**Figure S4.** **Comparison of the relative abundance of the high-mannose *N*-glycan structures with significant expressions** ((**a**) Man10, (**b**) Man7, and (**c**) Man5)) among different tissue cohorts. (*denotes *p-* value <0.05, **denotes *p-*value < 0.01). **^†^**N-glycan code: HexNAc_Hex_DeoxyHex_NeuAc_. Symbols:
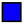
 , N-acetylglucosamine;
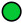
 , mannose.


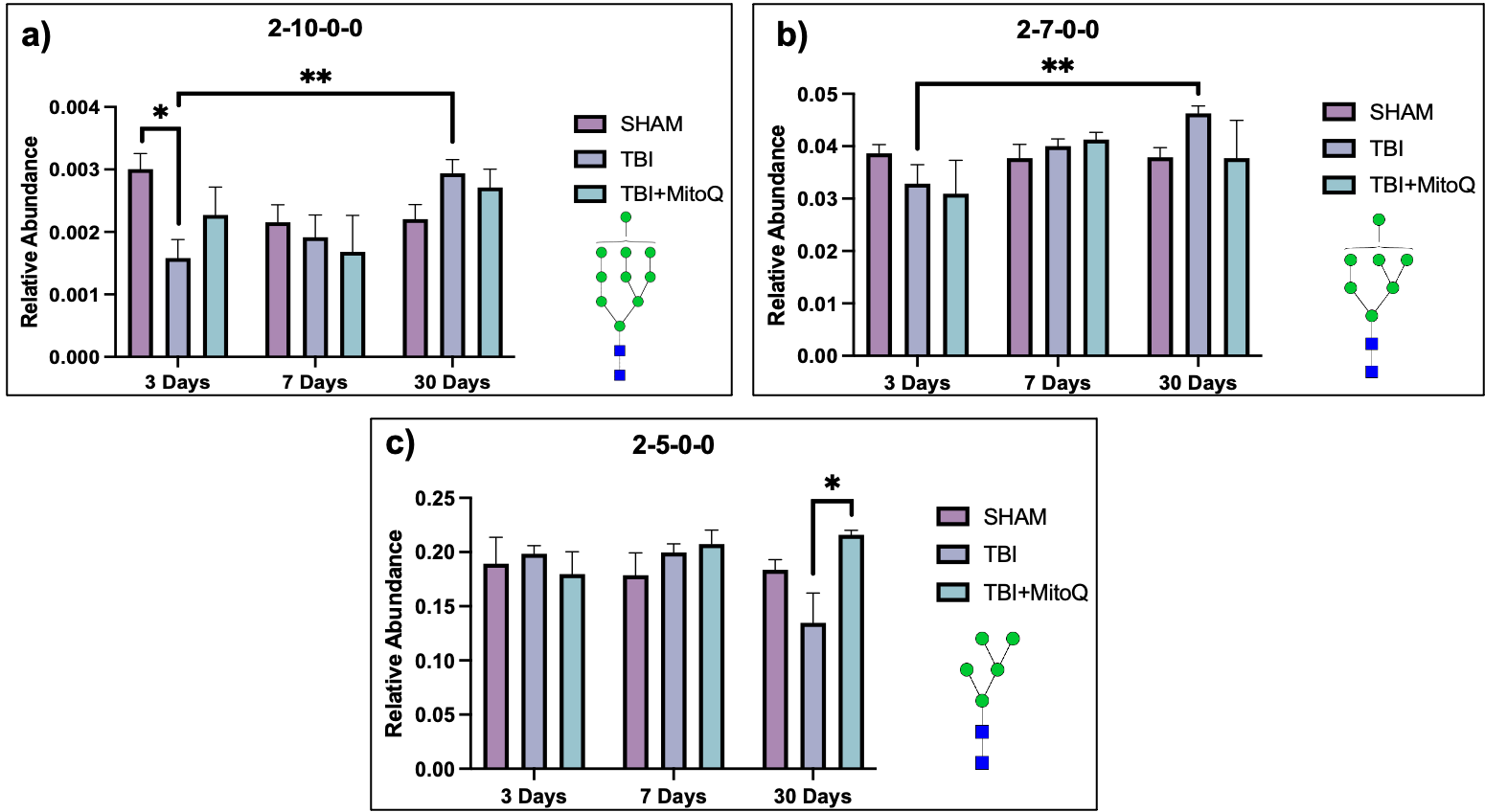


**Supporting Information Figure S5**

**Figure S5. Comparison of the relative abundance of the fucosylated *N*-glycan structures with significant expressions** ((**a**) HexNAc_4_Hex_4_DeoxyHex_1_, (**b**) HexNAc_4_Hex_5_DeoxyHex_3_, (**c**) HexNAc_4_Hex_6_DeoxyHex_2_, (**d**) HexNAc_5_Hex_3_DeoxyHex_1_, (**e**) HexNAc_5_Hex_4_DeoxyHex_2_, (**f**) HexNAc_6_Hex_4_DeoxyHex_1_, and (**g**) HexNAc_6_Hex_10_DeoxyHex_2_) among different tissue cohorts. (*denotes *p-* value <0.05, **denotes *p-*value < 0.01). **^†^**N-glycan code: HexNAc_Hex_DeoxyHex_NeuAc_. Symbols:
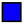
 , N-acetylglucosamine;
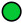
 , mannose;
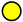
 , galactose;
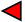
, fucose.

**
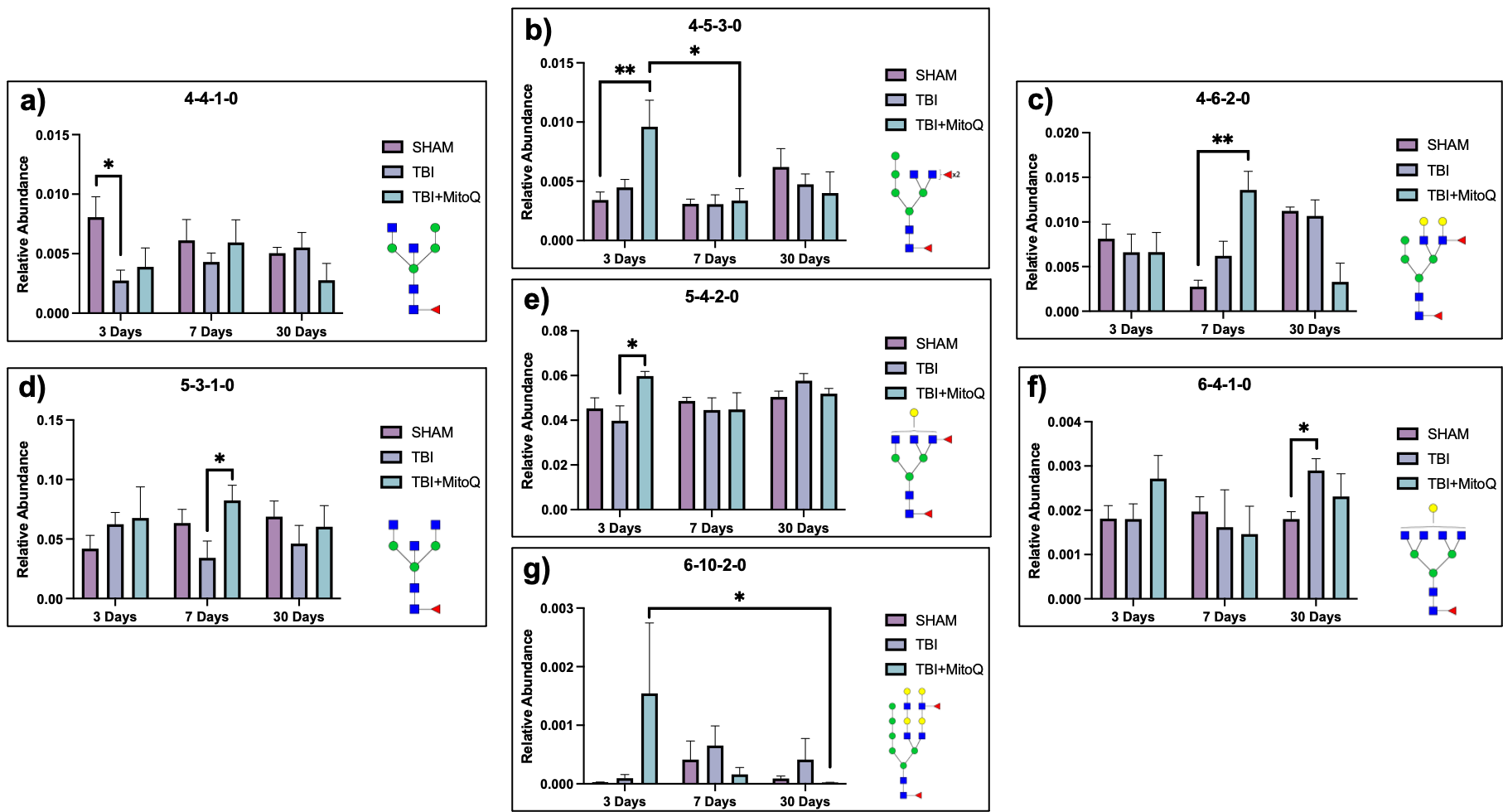
**

**Supporting Information Figure S6**

**Figure** **S6.** **Comparison of the relative abundance of the sialofucosylated *N*-glycan structures with significant expressions** ((**a**) HexNAc_6_Hex_6_DeoxyHex_3_NeuAc_1_, (**b**) HexNAc_6_Hex_7_DeoxyHex_3_NeuAc_2_, (**c**) HexNAc_4_Hex_5_DeoxyHex_3_NeuAc_1_, and (**d**) HexNAc_6_Hex_7_DeoxyHex_3_NeuAc_3_ among different tissue cohorts. (*denotes *p-* value <0.05). **^†^**N-glycan code: HexNAc_Hex_DeoxyHex_NeuAc_. Symbols:
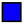
 , N-acetylglucosamine;
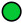
 , mannose;
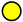
 , galactose;
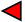
, fucose;
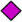
, N-acetylneuraminic acid.


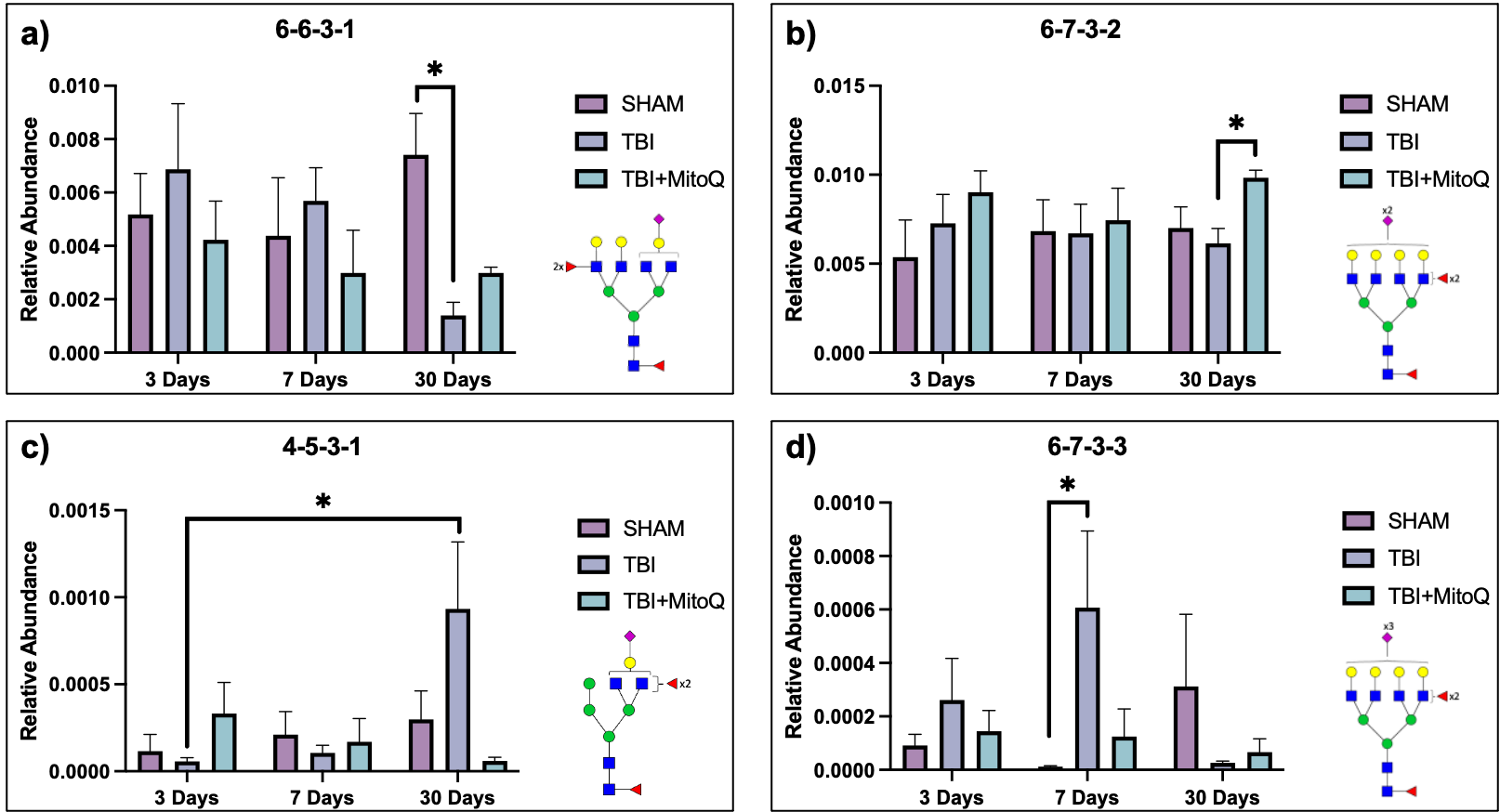


**Supporting Information Figure S7**

**Figure** **S7.** **Comparison of the relative abundance of other types of *N*-glycan structures with significant expressions** ((**a**) HexNAc_4_Hex_4_ and (**b**) HexNAc_4_Hex_5_) among different tissue cohorts. (*denotes *p-* value <0.05). **^†^**N-glycan code: HexNAc_Hex_DeoxyHex_NeuAc. Symbols:
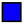
 , N-acetylglucosamine;
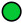
 , mannose;
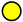
 , galactose.


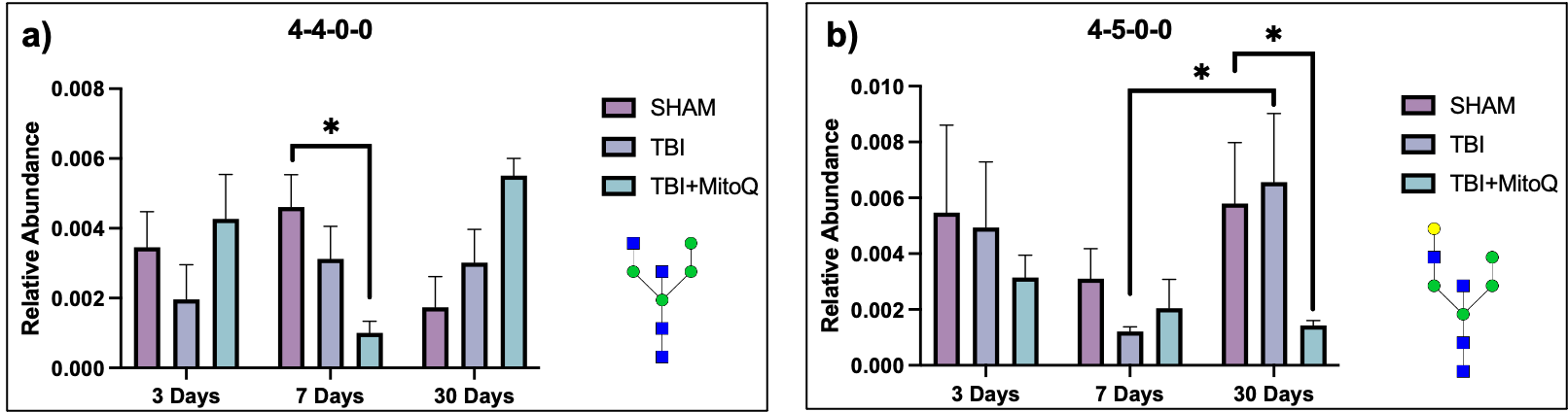

Supplement: Supplementary Figures [file neur.2025.0054_supplementary_figures.docx]
