# Supplementary material for: Exploring the Impact of Mitoquinone Supplementation on Glycan Profiles in a Repeated Mild Traumatic Brain Injury Mouse Model
Source: Neurotrauma Rep. 2025 Jun 16;6(1):525–38. doi: 10.1089/neur.2025.0054 (PMC12237849; doi:10.1089/neur.2025.0054)
Supplement: Supplementary Table S1 [file neur.2025.0054_supplementary_table_s1.pdf]

Table S1. N-Glycans profiling all brain tissue cohorts (SHAM, TBI, and TBI+MitoQ) at different stages (acute, subacute, and chronic time points) along with their average relative abundances and standard deviation. Four-digit code refers to the monosaccharide units, N-acetylglucosamine, Hexose, Fucose, and N-acetylneuraminic acid, respectively (HexNAc_Hex_DeoxyHex_NeuAc_).

	Code: HexNAc_Hex_DeoxyHex_NeuAc_	3 Days (Acute Stage)						7 Days (Subacute Stage)						30 Days (Chronic Time Points)					
	Glycan Conpositions	Ave. Rel. Abd. (Sham)	STDEV	Ave. Rel. Abd. (TBI)	STDEV	Ave. Rel. Abd. (TBI+MitoQ)	STDEV	Ave. Rel. Abd. (Sham)	STDEV	Ave. Rel. Abd. (TBI)	STDEV	Ave. Rel. Abd. (TBI+MitoQ)	STDEV	Ave. Rel. Abd. (Sham)	STDEV	Ave. Rel. Abd. (TBI)	STDEV	Ave. Rel. Abd. (TBI+MitoQ)	STDEV
High-mannose	2-3-0-0	2.56E-03	4.90E-04	2.17E-03	2.94E-04	2.04E-03	4.69E-04	2.26E-03	2.21E-04	2.06E-03	4.39E-04	2.09E-03	5.96E-04	1.92E-03	6.00E-04	2.71E-03	7.22E-04	1.79E-03	5.80E-04
	2-4-0-0	1.82E-03	7.47E-04	1.94E-03	3.02E-04	1.84E-03	1.21E-03	1.91E-03	1.01E-03	1.94E-03	6.78E-04	1.45E-03	1.37E-03	2.21E-03	1.50E-03	2.69E-03	1.03E-03	2.53E-03	8.92E-04
	2-5-0-0	1.89E-01	5.43E-02	1.98E-01	1.70E-02	1.79E-01	4.09E-02	1.78E-01	4.60E-02	1.99E-01	1.77E-02	2.07E-01	2.89E-02	1.84E-01	2.10E-02	1.35E-01	6.12E-02	2.16E-01	7.24E-03
	2-6-0-0	7.85E-02	2.62E-02	8.51E-02	4.83E-03	7.68E-02	1.85E-02	8.71E-02	9.51E-03	8.54E-02	7.10E-03	8.23E-02	2.55E-02	6.98E-02	2.17E-02	8.34E-02	1.48E-02	7.49E-02	2.78E-02
	2-7-0-0	3.87E-02	3.74E-03	3.29E-02	8.06E-03	3.09E-02	1.28E-02	3.77E-02	5.86E-03	4.00E-02	3.08E-03	4.13E-02	3.12E-03	3.79E-02	4.10E-03	4.63E-02	3.13E-03	3.77E-02	1.25E-02
	2-8-0-0	4.42E-02	8.02E-03	4.60E-02	4.04E-03	4.72E-02	5.16E-03	3.33E-02	1.75E-02	4.74E-02	2.26E-03	4.47E-02	2.06E-02	4.62E-02	4.12E-03	4.98E-02	1.62E-02	5.29E-02	1.49E-03
	2-9-0-0	1.84E-02	1.28E-02	2.91E-02	1.23E-02	3.85E-02	6.75E-03	3.12E-02	1.07E-02	3.24E-02	1.16E-02	3.27E-02	1.39E-02	3.95E-02	6.26E-03	3.65E-02	2.11E-02	3.46E-02	2.34E-02
	2-10-0-0	3.01E-03	5.54E-04	1.58E-03	6.66E-04	2.27E-03	8.96E-04	2.15E-03	6.30E-04	1.92E-03	8.01E-04	1.68E-03	1.31E-03	2.20E-03	5.25E-04	2.94E-03	4.92E-04	2.71E-03	5.13E-04
Fucosylated	2-3-1-0	7.83E-03	5.46E-03	5.71E-03	5.19E-03	6.80E-03	4.60E-03	6.54E-03	5.06E-03	4.42E-03	3.89E-03	7.87E-03	4.14E-03	7.73E-03	3.73E-03	1.09E-02	3.92E-03	4.81E-03	3.98E-03
	2-4-1-0	1.95E-03	1.59E-03	2.46E-03	1.37E-03	1.38E-03	8.24E-04	1.79E-03	1.41E-03	2.52E-03	1.08E-03	2.03E-03	1.48E-03	2.01E-03	1.31E-03	4.12E-04	3.58E-04	2.01E-03	1.69E-03
	2-5-1-0	7.23E-03	3.16E-03	7.77E-03	4.86E-04	8.05E-03	7.33E-04	8.78E-03	1.12E-03	8.05E-03	8.83E-04	7.95E-03	1.03E-03	7.84E-03	1.06E-03	8.75E-03	1.63E-03	8.43E-03	1.52E-03
	3-4-1-0	9.97E-03	3.22E-03	7.72E-03	4.23E-03	1.06E-02	3.00E-04	1.12E-02	1.12E-03	1.04E-02	8.70E-04	1.01E-02	1.09E-03	8.30E-03	3.17E-03	1.09E-02	1.59E-03	8.12E-03	5.89E-03
	3-5-1-0	1.36E-02	1.38E-03	1.24E-02	9.89E-04	1.35E-02	1.13E-03	1.23E-02	1.60E-03	1.23E-02	1.11E-03	1.31E-02	1.50E-03	1.11E-02	8.83E-04	1.30E-02	1.34E-03	1.24E-02	9.96E-04
	3-6-1-0	9.45E-03	3.90E-03	1.00E-02	1.66E-03	1.09E-02	1.17E-03	7.79E-03	3.98E-03	9.95E-03	6.84E-04	8.44E-03	4.63E-03	7.41E-03	4.02E-03	1.02E-02	4.23E-03	1.16E-02	1.95E-03
	3-7-1-0	4.05E-04	7.63E-04	4.87E-04	4.37E-04	8.33E-05	1.01E-04	3.43E-04	6.11E-04	5.86E-04	3.93E-04	1.04E-04	8.41E-05	1.90E-04	2.25E-04	8.66E-04	8.70E-04	1.55E-04	1.67E-04
	3-3-1-0	1.53E-02	3.33E-03	1.47E-02	1.90E-03	1.25E-02	2.36E-03	1.68E-02	2.58E-03	1.51E-02	1.88E-03	1.33E-02	4.51E-03	1.33E-02	3.78E-03	1.62E-02	6.41E-03	1.71E-02	6.92E-03
	3-4-2-0	1.30E-02	3.96E-03	1.38E-02	4.01E-03	6.30E-03	5.54E-03	1.70E-02	1.81E-03	1.28E-02	6.50E-03	7.20E-03	7.73E-03	1.10E-02	5.41E-03	1.05E-02	8.20E-03	8.45E-03	6.37E-03
	3-6-2-0	1.17E-02	1.24E-03	9.40E-03	2.47E-03	9.24E-03	4.89E-03	1.09E-02	1.44E-03	9.95E-03	1.44E-03	1.12E-02	8.76E-04	9.83E-03	5.91E-04	9.22E-03	5.15E-03	1.10E-02	2.21E-03
	4-4-1-0	8.06E-03	3.83E-03	2.75E-03	1.96E-03	3.89E-03	3.18E-03	6.13E-03	3.91E-03	4.30E-03	1.68E-03	5.94E-03	4.23E-03	5.04E-03	1.10E-03	5.52E-03	2.81E-03	2.77E-03	2.44E-03
	4-5-1-0	1.48E-02	4.14E-03	1.13E-02	2.94E-03	1.63E-02	8.47E-04	1.36E-02	2.03E-03	1.29E-02	6.44E-04	1.39E-02	4.57E-03	1.23E-02	9.49E-04	1.47E-02	2.72E-03	1.03E-02	5.30E-03
	4-5-2-0	8.60E-03	7.81E-03	9.01E-03	6.69E-03	8.92E-03	5.11E-03	5.38E-03	3.52E-03	4.63E-03	2.01E-03	6.88E-03	4.67E-03	4.29E-03	1.17E-03	4.86E-03	3.31E-03	3.08E-03	1.32E-03
	4-6-1-0	6.67E-03	3.16E-03	4.71E-03	1.76E-03	5.25E-03	2.45E-04	5.88E-03	2.60E-03	4.48E-03	2.09E-03	7.18E-03	3.78E-03	4.98E-03	1.90E-03	5.75E-03	2.78E-03	4.31E-03	6.30E-04
	4-6-2-0	8.14E-03	3.65E-03	6.62E-03	4.54E-03	6.63E-03	4.41E-03	2.77E-03	1.58E-03	6.22E-03	3.62E-03	1.36E-02	4.63E-03	1.12E-02	9.66E-04	1.07E-02	4.02E-03	3.29E-03	3.63E-03
	4-3-1-0	1.12E-01	1.67E-02	1.03E-01	7.45E-03	1.04E-01	7.96E-03	1.11E-01	1.07E-02	1.00E-01	1.15E-02	9.64E-02	2.99E-02	9.44E-02	1.36E-02	1.15E-01	1.92E-02	5.07E-02	3.92E-02
	4-4-2-0	3.16E-02	2.07E-02	3.84E-02	8.66E-03	4.07E-02	1.34E-02	4.12E-02	1.25E-02	4.63E-02	3.03E-03	2.65E-02	1.87E-02	4.43E-02	4.49E-03	3.74E-02	1.58E-02	5.02E-02	5.31E-03
	4-5-3-0	3.41E-03	1.51E-03	4.49E-03	1.50E-03	9.60E-03	4.47E-03	3.10E-03	8.75E-04	3.07E-03	1.76E-03	3.37E-03	2.24E-03	6.20E-03	3.45E-03	4.74E-03	1.95E-03	4.01E-03	3.09E-03
	4-6-3-0	4.49E-04	5.36E-04	7.18E-04	1.01E-03	9.76E-04	5.58E-04	3.58E-04	3.18E-04	2.45E-04	4.26E-04	8.58E-04	9.19E-04	3.38E-04	4.36E-04	9.58E-04	9.94E-04	3.27E-04	3.18E-04
	5-4-1-0	4.76E-03	2.39E-03	4.57E-03	2.32E-03	5.84E-03	1.18E-03	4.78E-03	3.10E-03	4.30E-03	2.01E-03	6.13E-03	1.04E-03	4.57E-03	1.93E-03	4.73E-03	2.12E-03	4.70E-03	3.01E-03
	5-5-2-0	3.56E-03	2.68E-03	3.18E-03	1.00E-03	3.52E-03	1.05E-03	2.91E-03	6.64E-04	3.62E-03	7.29E-04	4.56E-03	1.20E-03	2.45E-03	8.70E-04	3.40E-03	1.94E-03	3.18E-03	2.72E-04
	5-6-4-0	1.72E-02	8.66E-03	1.10E-02	6.52E-03	1.83E-02	6.92E-03	1.17E-02	7.14E-03	1.47E-02	4.85E-03	1.33E-02	1.17E-02	1.66E-02	1.04E-02	1.52E-02	1.33E-02	2.55E-02	3.98E-03
	5-9-1-0	1.02E-04	7.82E-05	9.55E-04	8.25E-04	8.08E-04	8.34E-04	7.17E-04	8.46E-04	6.55E-04	6.66E-04	4.21E-04	2.62E-04	8.46E-04	6.58E-04	2.72E-04	4.41E-04	1.68E-04	1.66E-04
	5-9-2-0	2.07E-04	2.18E-04	1.17E-04	1.03E-04	8.80E-05	5.04E-05	1.62E-04	2.82E-04	1.21E-04	1.91E-04	5.86E-05	4.74E-05	1.79E-04	1.91E-04	5.68E-05	5.20E-05	8.73E-05	1.28E-04
	5-3-1-0	4.19E-02	2.48E-02	6.24E-02	2.23E-02	6.76E-02	5.23E-02	6.35E-02	2.56E-02	3.42E-02	3.15E-02	8.24E-02	2.87E-02	6.88E-02	2.93E-02	4.62E-02	3.42E-02	6.03E-02	3.06E-02
	5-4-2-0	4.53E-02	1.06E-02	3.98E-02	1.48E-02	5.98E-02	4.23E-03	4.86E-02	3.65E-03	4.45E-02	1.23E-02	4.48E-02	1.68E-02	5.04E-02	5.83E-03	5.77E-02	7.16E-03	5.19E-02	4.08E-03
	5-5-1-0	1.63E-03	9.90E-04	8.86E-04	6.98E-04	7.32E-04	1.03E-03	6.60E-04	5.90E-04	1.30E-03	7.12E-04	7.01E-04	8.25E-04	1.21E-03	7.07E-04	5.91E-04	3.59E-04	2.74E-04	9.49E-05
	5-5-3-0	2.72E-02	1.56E-02	2.86E-02	1.27E-02	1.58E-02	1.35E-02	1.53E-02	1.35E-02	2.52E-02	1.82E-02	2.56E-02	1.80E-02	3.00E-02	1.50E-02	2.13E-02	1.85E-02	2.57E-02	1.52E-02
	5-6-2-0	2.07E-04	2.28E-04	1.35E-04	1.43E-04	1.49E-04	1.97E-04	2.50E-05	2.54E-05	3.95E-05	3.71E-05	1.54E-04	2.41E-04	4.51E-05	3.55E-05	1.55E-04	1.64E-04	2.28E-04	3.29E-04
	5-6-3-0	3.87E-04	2.37E-04	9.74E-04	5.71E-04	4.29E-04	5.31E-04	7.35E-04	9.34E-04	4.16E-04	5.18E-04	4.88E-04	5.60E-04	7.09E-04	5.65E-04	9.56E-04	1.60E-03	1.52E-03	2.31E-03
	6-3-1-0	9.74E-04	1.17E-03	2.20E-03	1.75E-03	3.31E-04	2.25E-04	2.69E-04	3.50E-04	6.08E-04	4.12E-04	3.49E-04	3.17E-04	2.66E-04	2.70E-04	4.36E-04	2.75E-04	4.70E-04	1.53E-04
	6-4-1-0	1.81E-03	6.57E-04	1.80E-03	7.70E-04	2.71E-03	1.05E-03	1.97E-03	7.45E-04	1.62E-03	1.89E-03	1.46E-03	1.41E-03	1.80E-03	3.79E-04	2.90E-03	5.96E-04	2.31E-03	8.93E-04
	6-5-2-0	6.02E-04	5.48E-04	5.66E-04	8.82E-04	2.94E-04	3.87E-04	4.31E-04	2.13E-04	8.89E-04	6.25E-04	2.12E-04	2.18E-04	4.82E-04	7.66E-04	9.89E-04	8.51E-04	1.02E-03	7.61E-04
	6-6-3-0	1.57E-03	2.65E-03	4.70E-04	5.07E-04	5.98E-04	4.88E-04	7.56E-04	3.88E-04	6.88E-04	7.99E-04	6.63E-04	8.50E-04	5.02E-04	4.80E-04	6.53E-04	6.14E-04	8.76E-04	7.71E-04
	6-7-4-0	1.29E-03	2.70E-03	1.92E-02	3.03E-02	2.34E-04	2.77E-04	2.41E-04	2.44E-04	1.52E-04	1.95E-04	3.82E-04	5.87E-04	2.40E-04	2.66E-04	1.80E-04	3.12E-04	2.07E-04	3.11E-04
	6-10-2-0	2.87E-05	7.21E-06	9.72E-05	1.39E-04	1.54E-03	2.40E-03	4.14E-04	7.04E-04	6.54E-04	7.47E-04	1.62E-04	2.62E-04	9.01E-05	9.86E-05	4.13E-04	8.05E-04	2.48E-05	4.54E-06
	6-4-2-0	4.92E-03	3.15E-03	2.51E-03	3.05E-03	2.52E-03	2.75E-03	2.72E-03	2.97E-03	3.48E-03	2.96E-03	2.13E-03	2.06E-03	1.09E-03	5.53E-04	6.89E-03	1.94E-03	2.88E-03	4.25E-03
	6-5-3-0	5.26E-03	2.50E-03	4.07E-03	2.45E-03	6.87E-03	9.31E-04	4.64E-03	1.25E-03	5.19E-03	1.59E-03	5.66E-03	3.80E-03	5.87E-03	2.09E-03	4.84E-03	3.68E-03	1.78E-03	5.15E-04
	6-6-4-0	3.78E-04	4.27E-04	1.03E-03	1.09E-03	1.48E-03	1.51E-03	9.10E-04	1.21E-03	3.62E-04	4.34E-04	2.82E-03	1.96E-03	7.32E-04	1.24E-03	6.34E-04	9.02E-04	2.78E-03	5.95E-04
	7-5-2-0	3.36E-05	2.54E-05	1.14E-04	2.02E-04	3.42E-05	1.55E-05	5.36E-05	2.34E-05	5.61E-05	2.43E-05	4.51E-05	2.71E-05	3.29E-05	2.34E-05	5.23E-05	2.69E-05	3.41E-05	7.34E-06
	7-9-1-0	2.29E-05	7.64E-06	8.54E-05	1.43E-04	5.06E-05	1.31E-05	3.21E-05	3.15E-05	8.70E-05	1.21E-04	2.55E-05	8.84E-06	1.10E-04	1.23E-04	4.97E-05	2.43E-05	2.46E-05	1.87E-05
	7-10-1-0	1.13E-04	1.26E-04	1.45E-04	2.03E-04	1.91E-04	1.58E-04	6.92E-05	7.19E-05	4.57E-04	6.85E-04	8.60E-05	6.18E-05	4.14E-04	6.20E-04	1.10E-04	7.25E-05	8.29E-05	6.04E-05
	7-10-2-0	1.19E-05	5.48E-06	5.44E-05	5.99E-05	3.11E-05	2.99E-05	3.75E-05	3.58E-05	2.23E-05	5.69E-06	3.26E-05	1.04E-05	2.20E-05	8.51E-06	3.04E-05	1.06E-05	3.82E-05	2.21E-05
	8-10-1-0	2.39E-05	1.13E-05	2.53E-05	2.20E-05	4.00E-05	2.48E-05	1.90E-05	9.90E-06	4.29E-05	2.58E-05	8.31E-05	1.33E-04	2.43E-05	1.32E-05	5.17E-05	3.51E-05	1.99E-05	1.12E-05
Sialylated	3-5-0-2	1.54E-03	7.40E-04	4.87E-04	3.65E-04	1.34E-03	7.66E-04	6.06E-04	4.13E-04	7.46E-04	4.50E-04	5.41E-04	5.17E-04	5.65E-04	3.22E-04	5.28E-04	4.18E-04	1.28E-03	1.73E-03
	3-6-0-1	7.49E-03	3.54E-03	7.59E-03	2.09E-03	8.16E-03	1.91E-03	8.78E-03	1.40E-03	8.42E-03	1.10E-03	6.54E-03	3.75E-03	7.45E-03	6.81E-04	7.22E-03	4.74E-03	6.42E-03	5.06E-03
	4-5-0-1	1.45E-03	1.66E-03	1.13E-03	8.58E-04	2.32E-03	1.94E-03	2.95E-03	1.83E-03	4.06E-03	3.49E-03	2.60E-03	2.38E-03	2.27E-03	1.88E-03	1.96E-03	2.21E-03	3.34E-03	2.00E-03
	4-6-0-1	8.47E-04	9.65E-04	2.38E-04	2.46E-04	3.54E-04	4.00E-04	5.97E-04	6.56E-04	1.58E-03	1.23E-03	8.62E-04	8.65E-04	7.08E-04	7.16E-04	1.75E-03	1.76E-03	4.38E-04	4.87E-04
	4-6-0-2	2.29E-04	3.80E-04	1.36E-04	1.59E-04	3.38E-04	4.43E-04	3.96E-04	3.53E-04	1.99E-04	2.55E-04	2.02E-04	2.98E-04	1.19E-04	1.27E-04	2.72E-04	3.39E-04	3.07E-05	7.51E-06
	3-5-1-1	5.31E-03	2.38E-03	5.17E-03	6.80E-04	3.64E-03	2.47E-03	3.48E-03	2.34E-03	3.28E-03	2.36E-03	3.47E-03	1.80E-03	4.31E-03	2.69E-03	3.74E-03	2.09E-03	3.83E-03	3.00E-03

Sialylated & Fucosylated	3-6-1-1	3.80E-03	4.04E-03	4.53E-03	2.75E-03	3.02E-03	2.89E-03	8.01E-04	6.55E-04	2.66E-03	3.09E-03	2.06E-03	2.44E-03	7.74E-04	3.01E-04	9.47E-04	1.29E-03	1.67E-03	1.54E-03
	3-6-1-2	2.34E-03	9.36E-04	1.80E-03	1.17E-03	1.50E-03	1.12E-03	1.74E-03	9.46E-04	1.78E-03	1.22E-03	1.31E-03	1.56E-03	1.57E-03	1.09E-03	1.83E-03	1.45E-03	1.62E-03	1.12E-03
	4-4-1-2	2.31E-03	1.22E-03	2.26E-03	1.27E-03	2.36E-03	9.33E-04	2.39E-03	1.63E-03	2.89E-03	4.57E-04	2.15E-03	8.56E-04	1.10E-03	1.10E-03	2.73E-03	3.56E-04	1.49E-03	1.54E-03
	4-5-1-1	1.40E-03	1.02E-03	1.88E-03	1.42E-03	5.00E-03	5.13E-03	3.60E-03	3.42E-03	2.03E-03	6.10E-04	3.01E-03	1.07E-03	3.75E-03	3.58E-03	2.90E-03	2.22E-03	2.09E-03	2.27E-03
	4-5-1-2	6.04E-04	8.46E-04	8.38E-04	6.78E-04	3.13E-04	3.14E-04	7.37E-04	3.58E-04	1.01E-03	7.21E-04	4.57E-04	5.39E-04	6.25E-04	3.25E-04	7.35E-04	6.78E-04	1.36E-03	1.28E-03
	4-5-1-3	1.69E-03	1.27E-03	1.34E-03	8.19E-04	7.04E-04	1.09E-03	1.24E-03	1.03E-03	1.64E-03	4.45E-04	1.40E-03	1.18E-03	7.58E-04	5.55E-04	1.07E-03	5.82E-04	1.50E-03	9.14E-04
	4-5-2-1	3.70E-03	3.06E-03	3.54E-03	3.98E-03	1.25E-03	9.53E-04	8.51E-04	3.14E-04	2.14E-03	1.21E-03	1.11E-03	9.54E-04	2.05E-03	1.58E-03	1.23E-03	9.54E-04	1.19E-03	1.35E-03
	4-5-2-2	3.34E-03	2.32E-03	3.32E-03	2.67E-03	8.78E-04	6.57E-04	3.49E-03	3.34E-03	4.06E-03	3.40E-03	2.55E-03	3.66E-03	5.64E-03	2.47E-03	2.37E-03	2.82E-03	5.76E-03	4.11E-03
	4-5-3-1	1.16E-04	2.13E-04	5.70E-05	4.81E-05	3.32E-04	3.57E-04	2.11E-04	2.94E-04	1.06E-04	9.71E-05	1.70E-04	2.99E-04	2.98E-04	3.67E-04	9.34E-04	8.62E-04	5.93E-05	3.77E-05
	4-6-1-1	5.98E-04	5.93E-04	7.39E-04	4.55E-04	4.06E-04	1.91E-04	4.55E-04	4.82E-04	3.96E-04	1.80E-04	5.54E-04	1.25E-04	2.82E-04	2.31E-04	3.52E-04	3.64E-04	2.63E-04	2.30E-04
	4-6-2-1	2.56E-04	2.72E-04	5.63E-04	5.79E-04	7.80E-05	1.02E-04	6.75E-04	7.14E-04	5.19E-04	4.57E-04	1.24E-04	9.56E-05	2.81E-04	2.76E-04	5.14E-04	4.18E-04	3.97E-04	4.97E-04
	5-4-1-1	5.60E-03	3.35E-03	5.67E-03	2.83E-03	4.89E-03	4.66E-03	5.10E-03	2.44E-03	4.57E-03	3.29E-03	6.40E-03	4.86E-03	5.26E-03	3.68E-03	5.57E-03	4.17E-03	2.58E-03	7.11E-04
	5-5-2-1	1.24E-03	8.04E-04	1.83E-03	1.04E-03	1.40E-03	3.56E-04	2.10E-03	2.37E-03	1.27E-03	1.04E-03	1.27E-03	7.24E-04	9.53E-04	6.69E-04	2.07E-03	1.90E-03	1.80E-03	2.05E-03
	5-6-1-1	1.88E-04	2.23E-04	4.20E-04	3.72E-04	4.65E-04	3.84E-04	7.84E-05	3.90E-05	2.98E-04	2.75E-04	1.07E-04	1.15E-04	2.46E-04	2.47E-04	2.76E-04	3.37E-04	6.17E-05	3.30E-05
	5-6-2-1	6.22E-04	1.19E-03	9.41E-04	1.16E-03	2.96E-04	2.64E-04	6.88E-04	8.92E-04	6.48E-04	1.23E-03	1.46E-03	1.67E-03	1.44E-03	1.94E-03	8.09E-04	1.50E-03	1.77E-03	2.91E-03
	5-6-2-2	9.21E-04	1.14E-03	8.75E-04	9.37E-04	5.78E-04	3.74E-04	9.23E-04	1.15E-03	5.74E-04	5.58E-04	2.01E-04	1.39E-04	5.04E-04	3.55E-04	4.47E-04	3.00E-04	7.08E-04	1.02E-03
	5-6-1-3	7.79E-04	5.88E-04	1.41E-04	1.91E-04	2.95E-04	3.86E-04	7.49E-04	7.91E-04	3.21E-04	2.78E-04	3.47E-04	6.19E-04	5.40E-04	8.25E-04	5.33E-04	5.67E-04	8.74E-04	1.44E-03
	5-6-3-2	1.33E-03	8.22E-04	8.93E-04	6.77E-04	1.18E-03	1.05E-03	9.69E-04	8.28E-04	1.06E-03	8.81E-04	8.98E-04	1.01E-03	1.08E-03	8.52E-04	6.09E-04	7.33E-04	2.00E-03	4.75E-04
	6-4-1-1	1.63E-03	1.61E-03	1.17E-03	8.00E-04	1.69E-03	8.41E-04	3.46E-03	4.58E-03	2.07E-03	1.39E-03	9.53E-04	1.10E-03	2.00E-03	8.29E-04	1.74E-03	1.88E-03	3.38E-03	3.32E-03
	6-5-1-1	2.63E-04	2.13E-04	3.88E-04	5.20E-04	3.05E-04	3.74E-04	2.26E-04	3.94E-04	4.49E-04	6.61E-04	1.24E-04	1.19E-04	2.47E-04	4.66E-04	2.83E-04	2.88E-04	5.07E-04	6.38E-04
	6-5-1-2	1.26E-03	2.40E-03	9.31E-04	1.02E-03	1.77E-04	9.31E-05	8.04E-04	9.88E-04	9.90E-05	1.17E-04	2.18E-04	2.90E-04	2.69E-04	1.79E-04	3.43E-04	6.19E-04	7.54E-05	2.77E-05
	6-5-2-1	4.09E-03	3.02E-04	3.94E-03	6.21E-04	3.43E-03	1.36E-03	4.39E-03	2.96E-03	4.34E-03	1.60E-03	5.41E-03	3.74E-03	5.00E-03	3.23E-03	4.14E-03	1.82E-03	4.38E-03	1.22E-04
	6-6-2-1	1.08E-04	1.76E-04	9.99E-05	8.25E-05	2.67E-05	2.37E-05	8.51E-05	8.67E-05	6.59E-05	6.31E-05	4.01E-05	1.88E-05	5.08E-05	4.43E-05	5.28E-05	5.58E-05	4.54E-04	7.30E-04
	6-5-2-2	1.35E-02	2.36E-02	2.58E-03	2.78E-03	1.52E-02	1.41E-02	2.20E-02	3.80E-02	2.21E-02	3.57E-02	3.75E-03	3.54E-03	2.24E-02	3.01E-02	2.19E-02	4.05E-02	8.19E-03	4.49E-03
	6-6-2-2	8.63E-04	1.40E-03	8.66E-04	4.65E-04	2.29E-03	2.00E-03	1.71E-03	1.94E-03	1.78E-03	4.91E-04	1.79E-03	8.98E-04	1.29E-03	9.37E-04	2.54E-03	4.45E-03	2.75E-03	2.55E-03
	6-6-3-1	5.17E-03	3.41E-03	6.86E-03	5.49E-03	4.23E-03	2.90E-03	4.37E-03	4.86E-03	5.69E-03	2.77E-03	2.99E-03	3.57E-03	7.41E-03	3.46E-03	1.40E-03	1.09E-03	2.99E-03	3.79E-04
	6-6-3-2	5.31E-03	5.42E-03	5.52E-03	3.93E-03	6.78E-03	3.55E-03	6.28E-03	3.11E-03	8.01E-03	1.85E-03	3.66E-03	4.24E-03	3.39E-03	3.27E-03	5.50E-03	4.64E-03	1.09E-02	4.86E-04
	6-7-1-4	2.98E-04	6.19E-04	4.85E-04	6.48E-04	9.51E-05	1.39E-04	4.00E-04	5.37E-04	9.15E-05	1.34E-04	1.78E-05	8.29E-06	4.73E-05	4.71E-05	2.45E-05	1.71E-05	7.42E-05	8.38E-05
	6-7-2-2	3.76E-03	6.18E-03	2.67E-03	3.63E-03	1.86E-03	8.95E-04	5.29E-03	5.46E-03	2.49E-03	4.56E-03	2.47E-03	4.96E-03	1.71E-03	1.71E-03	6.42E-03	7.17E-03	9.47E-04	2.01E-04
	6-7-2-3	1.30E-03	1.16E-03	1.24E-03	7.60E-04	1.38E-03	1.11E-03	2.28E-03	1.76E-03	1.87E-03	1.35E-03	1.51E-03	1.39E-03	7.57E-04	7.68E-04	9.14E-04	6.76E-04	3.96E-04	3.32E-04
	6-7-3-1	8.54E-04	6.04E-04	7.97E-04	6.20E-04	4.90E-04	2.53E-04	3.17E-04	2.34E-04	2.20E-04	3.57E-04	1.20E-03	8.48E-04	6.90E-04	8.16E-04	1.00E-03	8.06E-04	1.14E-03	9.66E-04
	6-7-3-2	5.37E-03	4.66E-03	7.27E-03	3.63E-03	9.02E-03	2.42E-03	6.83E-03	3.91E-03	6.70E-03	3.66E-03	7.46E-03	4.02E-03	7.01E-03	2.65E-03	6.15E-03	1.87E-03	9.83E-03	7.48E-04
	6-7-3-3	9.10E-05	9.39E-05	2.61E-04	3.50E-04	1.45E-04	1.56E-04	1.24E-05	6.96E-06	6.07E-04	6.42E-04	1.25E-04	2.30E-04	3.12E-04	6.03E-04	2.59E-05	1.52E-05	6.61E-05	8.75E-05
	6-7-4-1	9.51E-03	4.51E-03	4.59E-03	3.34E-03	3.09E-03	3.31E-03	4.67E-03	3.79E-03	5.29E-03	2.59E-03	3.68E-03	4.27E-03	3.74E-03	2.91E-03	6.21E-03	3.91E-03	8.17E-03	4.40E-03
	7-5-2-1	7.38E-04	8.39E-04	5.50E-04	1.79E-04	6.70E-04	7.11E-04	5.22E-04	4.27E-04	1.14E-03	4.46E-04	1.11E-03	8.04E-04	1.33E-03	6.31E-04	6.11E-04	7.00E-04	1.39E-03	1.51E-03
	7-6-2-2	9.56E-04	7.47E-04	1.09E-03	7.94E-04	3.17E-04	2.57E-04	6.59E-04	2.96E-04	6.97E-04	6.83E-04	3.55E-04	2.84E-04	7.37E-04	8.05E-04	2.12E-04	1.15E-04	1.93E-03	1.46E-03
	7-6-3-1	1.92E-03	1.15E-03	2.82E-03	1.93E-03	1.93E-03	1.74E-03	3.59E-03	1.62E-03	3.32E-03	1.80E-03	1.64E-03	3.58E-04	4.53E-03	1.45E-03	3.83E-03	3.60E-03	3.87E-03	2.36E-03
	7-8-5-1	6.07E-04	6.42E-04	1.64E-04	2.31E-04	6.84E-04	6.05E-04	3.23E-04	4.34E-04	3.15E-04	4.45E-04	9.32E-05	1.07E-04	1.62E-04	2.46E-04	4.74E-04	4.66E-04	3.47E-04	4.71E-04
Other Structures	3-3-0-0	1.64E-03	4.33E-04	1.46E-03	1.83E-04	7.96E-04	8.23E-04	1.56E-03	6.92E-04	1.66E-03	2.59E-04	9.81E-04	8.17E-04	1.36E-03	4.22E-04	1.91E-03	7.79E-04	1.55E-03	1.42E-03
	3-4-0-0	6.89E-03	5.88E-03	5.45E-03	6.99E-03	2.40E-03	2.46E-03	4.39E-03	3.43E-03	4.21E-03	3.98E-03	1.50E-03	1.31E-03	2.04E-03	8.12E-04	1.79E-03	1.12E-03	2.88E-03	1.43E-03
	3-5-0-0	1.33E-02	1.38E-03	1.23E-02	1.41E-03	1.33E-02	6.24E-04	1.36E-02	1.70E-03	1.26E-02	7.22E-04	1.28E-02	1.30E-03	1.23E-02	1.50E-03	1.37E-02	2.15E-03	1.38E-02	2.39E-03
	3-6-0-0	2.23E-03	4.90E-04	1.61E-03	8.03E-04	2.24E-03	2.84E-04	2.14E-03	3.93E-04	1.92E-03	1.89E-04	2.32E-03	4.59E-04	2.00E-03	2.68E-04	2.31E-03	4.26E-04	2.24E-03	4.61E-04
	4-3-0-0	1.54E-02	3.49E-03	1.14E-02	5.53E-03	1.22E-02	2.02E-03	1.61E-02	2.78E-03	1.43E-02	2.08E-03	1.17E-02	4.81E-03	1.27E-02	3.04E-03	1.63E-02	5.18E-03	1.66E-02	6.82E-03
	4-4-0-0	3.45E-03	2.29E-03	1.96E-03	2.22E-03	4.27E-03	2.54E-03	4.61E-03	2.08E-03	3.12E-03	2.09E-03	1.00E-03	7.58E-04	1.74E-03	1.97E-03	3.02E-03	2.12E-03	5.51E-03	8.53E-04
	4-5-0-0	5.46E-03	6.98E-03	4.94E-03	5.25E-03	3.14E-03	1.60E-03	3.10E-03	2.41E-03	1.21E-03	3.79E-04	2.04E-03	2.32E-03	5.79E-03	4.87E-03	6.56E-03	5.50E-03	1.43E-03	3.05E-04
	4-7-0-0	9.26E-05	1.50E-04	2.19E-04	3.39E-04	1.70E-04	1.90E-04	1.63E-04	2.17E-04	4.22E-04	4.61E-04	9.11E-05	1.80E-04	1.44E-04	1.14E-04	5.94E-05	5.98E-05	9.72E-05	6.37E-05
	5-3-0-0	2.63E-02	6.46E-03	2.17E-02	3.03E-03	2.10E-02	1.13E-02	2.35E-02	7.22E-03	2.38E-02	3.81E-03	2.63E-02	4.60E-03	2.32E-02	3.02E-03	3.00E-02	7.80E-03	2.79E-02	9.26E-03
	5-4-0-0	2.91E-04	3.87E-04	7.20E-04	9.10E-04	6.06E-04	8.78E-04	4.54E-04	2.45E-04	7.98E-04	4.38E-04	4.46E-04	5.65E-04	3.90E-04	4.22E-04	3.14E-04	4.05E-04	1.21E-03	7.35E-04
